# Sequence‐Based Multi Ancestry Association Study Reveals the Polygenic Architecture of *Varroa destructor* Resistance in the Honeybee *Apis mellifera*

**DOI:** 10.1111/mec.17637

**Published:** 2024-12-31

**Authors:** Sonia E. Eynard, Fanny Mondet, Benjamin Basso, Olivier Bouchez, Yves Le Conte, Benjamin Dainat, Axel Decourtye, Lucie Genestout, Matthieu Guichard, François Guillaume, Emmanuelle Labarthe, Barbara Locke, Rachid Mahla, Joachim de Miranda, Markus Neuditschko, Florence Phocas, Kamila Canale‐Tabet, Alain Vignal, Bertrand Servin

**Affiliations:** ^1^ GenPhySE Université de Toulouse, INRAE, ENVT Castanet‐Tolosan France; ^2^ LABOGENA DNA Palaiseau France; ^3^ UMT PrADE Avignon France; ^4^ INRAE, UR 406 Abeilles et Environnement Avignon France; ^5^ ITSAP Avignon France; ^6^ GeT‐PlaGe, Genotoul INRAE Castanet‐Tolosan France; ^7^ Agroscope Swiss Bee Research Centre Bern Switzerland; ^8^ Animal GenoPhenomics, Agroscope Posieux Switzerland; ^9^ SYNETICS Noyal‐sur‐Vilaine France; ^10^ Department of Ecology Swedish University of Agricultural Sciences Uppsala Sweden; ^11^ INRAE, AgroParisTech, GABI Université Paris‐Saclay Jouy‐en‐Josas France

**Keywords:** *Apis mellifera*, GWAS, host–parasite interaction, mite non reproduction, pool sequencing, recapping, *Varroa destructor*, varroa resistance

## Abstract

Honeybees, *Apis mellifera* , have experienced the full impacts of globalisation, including the recent invasion by the parasitic mite *Varroa destructor* , now one of the main causes of colony losses worldwide. The strong selection pressure it exerts has led some colonies to develop defence strategies conferring some degree of resistance to the parasite. Assuming these traits are partly heritable, selective breeding of naturally resistant bees could be a sustainable strategy for fighting infestations. To characterise the genetic determinism of varroa resistance, we conducted the largest genome wide association study performed to date on whole genome sequencing of more than 1500 colonies on multiple phenotypes linked to varroa resistance of honeybees. To take into account some genetic diversity of honeybees, colonies belonging to different ancestries representing the main honeybee subspecies in Western Europe were included and analysed both as separate populations and combined in a meta‐analysis. The results show that varroa resistance is substantially heritable and polygenic: while 60 significant associations were identified, none explain a substantial part of the trait genetic variance. Overall our study highlights that genomic selection for varroa resistance is promising but that it will not be based on managing a few strong effect mutations and rather use approaches that leverage the genome wide diversity of honeybee populations. From a broader perspective, these results point the way towards understanding the genetic adaptation of eusocial insects to parasite load.

## 1 Introduction

The honeybee, *Apis mellifera* , is a crucial contributor to sustainable food production (Klein et al. 2007). However, beekeepers have been experiencing dramatic colony losses for the past two decades (Bruckner et al. 2023; Potts et al. 2010). Such losses are sustainable neither for beekeepers nor for the agroecosystems relying on services provided by honeybees. Beekeepers themselves are major actors to counter such losses, through knowledge acquisition on the biology of honeybees, on threats they face and on good beekeeping practices (Jacques et al. 2017). Extensive research has shown that honeybees are threatened by multiple factors: both abiotic, with the loss of natural resources and the impact of pesticides due to agriculture intensification; and biotic, with the infection by a diversity of pests and parasites that impair bee survival (Goulson et al. 2015). Among biotic factors, the ectoparasite *Varroa destructor* is currently considered as the main threat to honeybee health and beekeeping worldwide (Traynor et al. 2020). In most regions of the world, colony losses have dramatically increased since its introduction in *Apis mellifera* populations in the 1960s (Eastern Europe) and the 1970s (Western Europe) (Le Conte, Ellis, and Ritter 2010; Traynor et al. 2020). Originating from Asia, where a stable host–parasite relationship exists with its historical host *Apis cerana* , varroa now infests most *Apis mellifera* colonies worldwide. Varroa invades multiple compartments of the honeybee colony: it reproduces in the brood, feeds on adult honeybee haemolymph and fat body, and is associated with a higher prevalence of viral infections (de Miranda et al. 2011; Rosenkranz, Aumeier, and Ziegelmann 2010; Traynor et al. 2020). Combined together, these effects on individual bees can lead to colony collapse within a few months if no actions are taken to control mite infestations (Fries, Imdorf, and Rosenkranz 2006). To date, managing varroa infestation presents many constraints, offering beekeepers only a few unsustainable solutions to fight the deadly mite (Rosenkranz, Aumeier, and Ziegelmann 2010; Noël, Le Conte, and Mondet 2020). Some rely on the use of chemical compounds, such as amitraz and fluvalinate. These must be administered under strict veterinarian prescription and are banned from organic farming. In addition, there has been a rise in varroa resistance to these chemicals over recent years (Rinkevich 2020).

Since the beginning of the 1990's, colonies naturally surviving varroa infestation without treatment have been observed in several regions of the world, raising hope for beekeepers to overcome the problem. Such populations have been identified in the USA, France, Sweden, Norway and South America (Mondet, Beaurepaire, et al. 2020). In these surviving colonies, honeybees often display behavioural defences against the parasite. For example, honeybees are observed cleaning brood frames or grooming each other more extensively (Dadoun et al. 2020; Spivak and Danka 2020). These collective responses might contribute to the limitation of parasite population growth and could provide colonies with social immunity (Cremer, Armitage, and Schmid‐Hempel 2007), a sustainable long‐term adaptation to counter the immense damage caused by varroa. The defence repertoire against varroa includes: (i) hygienic behaviour, the non‐specific cleaning of damaged brood cells, (ii) varroa sensitive hygiene (VSH; Harbo and Harris 2005), the targeted cleaning of specific cells parasitised by varroa, or (iii) recapping behaviour (Mondet, Beaurepaire, et al. 2020; Mondet, Parejo, et al. 2020; Oddie, Dahle, and Neumann 2018), consisting in removing the cap of brood cells followed by recapping, and (iv) a physiological mechanisms called suppressed mite reproduction (SMR; Mondet, Beaurepaire, et al. 2020; Harbo and Harris 1999), which describes a disruption in mite reproduction due to an action of the brood itself. Expression of these different traits lead to an increase in mite non reproduction within brood cells (MNR; Mondet, Parejo, et al. 2020; Harbo and Harris 1999), also called decreased mite reproduction, DMR (von Virag et al. 2022). These are associated with lower overall mite population and limit growth and infestation (Noël, Le Conte, and Mondet 2020). Such mechanisms have raised interest within the beekeeping sector as well as academic interest in deciphering the genetic mechanisms underlying varroa resistance in the honeybee.

As for common livestock species, the most straightforward way to increase varroa resistance in the honeybee population appears to be through selection and dissemination of the most resistant lines. Since the late 1990s, efforts have been put into the selective breeding of such resistant honeybee strains (Rinderer et al. 2010; Büchler et al. 2020). Yet, selecting for complex traits in honeybees has been hindered by features that strongly differentiate honeybees from other more typical livestock species. Due to the social nature of honeybees, many phenotypic traits of interest to beekeeping (including varroa resistance traits) are expressed at the group (i.e., colony) level and thus cannot be addressed by classical GWAS approaches where phenotypes are determined at the individual level. In addition, many of these traits are linked to group behaviours and are thus difficult to phenotype and can display rather low repeatabilities and heritabilities (Rothenbuhler 1964; Bienefeld and Pirchner 1990; Harbo and Harris 2009; Eynard et al. 2020; Guichard et al. 2020).

At the breeding level, managing honeybee reproduction can be difficult. Honeybees are polyandric (Tarpy, Hatch, and Fletcher 2000), meaning that one queen mates with multiple males. In nature, this mating happens once in the honeybee queen's life. Typically, a honeybee queen can fly multiple kilometres to reach a male congregation and mate with up to 20 males, storing sperm in her spermatheca. This will be used throughout the queen's life to produce diploid organisms, female worker bees or daughter queens. Contrarily, male offspring or drones, are born from unfertilised eggs and are consequently haploid. In addition, sex determination is governed by a haplodiploid mechanism at a single locus, the complementary sex determiner (*CSD*) locus (Zayed 2004; Beye et al. 2003) at which diploid homozygosity is lethal (Woyke 1963), limiting inbreeding drastically. Finally, honeybee populations bred for beekeeping encompass an important genetic diversity, with strong regional clustering, which impedes the identification of genetic markers that are relevant outside the population where they are identified. Developing molecular tools to assess the genetic make‐up of honeybee colonies could help in honeybee breeding by coping with some of these issues. To list a few possibilities, genomic‐enabled prediction of resistance traits could reduce the amount of complex phenotyping required by identifying promising colonies early in life. Also, genetic assessment could help identify the genetic structure of colonies (Eynard et al. 2022), allowing the design of crosses limiting inbreeding in selection programmes.

In addition to their use in selection programmes, molecular tools can help to identify pathways involved in resistance by assessing the statistical effects of polymorphisms on the variability of complex phenotypes, using genome wide association studies (GWAS). This offers opportunities to develop new approaches that take into account the specific genetic determinism of honeybees and to open avenues for genomic selection on traits such as varroa resistance. However, tools to perform genetic association studies are so far not tailored to encompass the genetic specificities of honeybees, limiting the power of genomic studies performed on this species and their transferability into breeding tools. Some markers associated with varroa resistance have been identified (see Mondet, Beaurepaire, et al. 2020 for a review) but most genomic studies performed on honeybee traits so far were built on a limited number of samples (10–200 individuals or colonies; Avalos et al. 2020; Shorter et al. 2012; Liu et al. 2016; Southey et al. 2016; Conlon et al. 2019; Guichard, Dainat, et al. 2021; Guichard et al. 2021; Guichard et al. 2022), restricting the power of the analyses and their use for developing a general breeding tool. To date, the use of potential markers has been limited, mostly due to a lack of low‐cost genotyping tools, leaving beekeepers with very limited access to varroa resistant stock.

In this study, we took advantage of the unique situation represented by the French honeybee populations. Indeed, the French territory has the advantage of presenting a large variety of landscapes, environments and ecosystems, where honeybee populations with different genetic backgrounds coexist, together with a large variety of hybrid colonies (Wragg et al. 2022). To take this diversity into account, we performed one of the largest genomic studies to date applied to honeybees, with the phenotyping and complete genome sequencing of more than 1500 colonies. Using uniquely tailored genetic and genomic tools, such as queen genotype reconstruction from pool sequence data (Eynard et al. 2022), GWAS and meta‐GWAS analyses (Morris 2011; Urbut et al. 2019), we investigated the genetic basis of three major traits linked to varroa resistance: overall varroa infestation of the colony, MNR and recapping of varroa infested brood cells. We identified multiple genetic markers associated with these traits on several chromosomes and evidenced the heterogeneity of the genomic regions involved across populations. This large‐scale effort provides a new understanding of the genetic mechanism underlying honeybee resistance to its main parasite, the *Varroa destructor* mite.

## 2 Materials and Methods

### 2.1 Honeybee Colonies and Sampling Strategy

**FIGURE 1:**
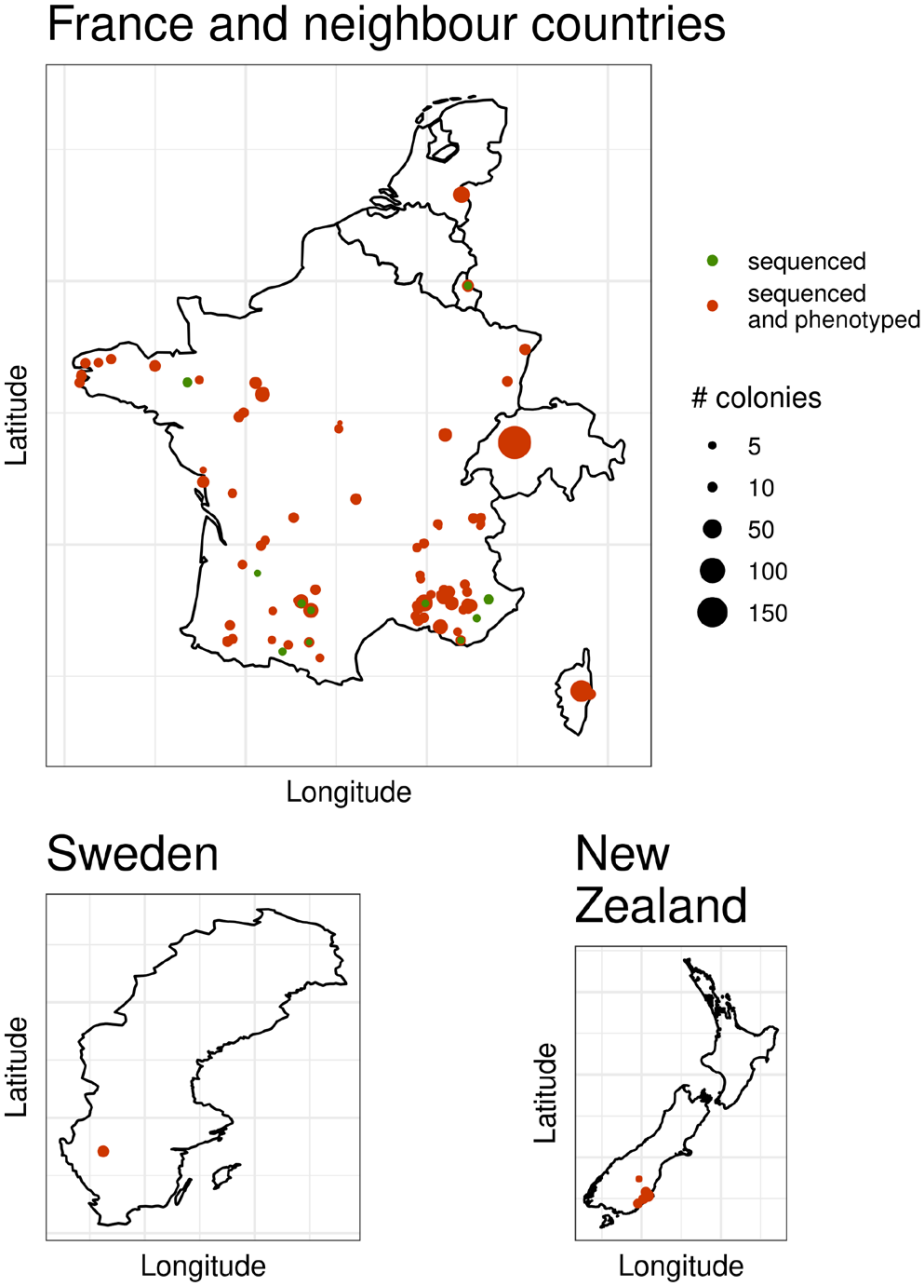
Geographic distribution of the sampled colonies. Geographical locations of colonies that were only sequenced (in green) and both phenotyped and sequenced (in red). The size of the dot represents the number of honeybee colonies per location and category.

The sampling strategy was established to represent the diversity (both in terms of genetic background and beekeeping practices) of honeybee colonies bred in Western Europe, mostly in France, but also including several other European and non‐European countries, to enlarge our sampling base. A total of 97 beekeepers participated in this study. They were located mostly in France but also in Switzerland, Luxembourg, the Netherlands, Sweden and New Zealand (Figure 1). The colonies outside France were included as they show similar genetic backgrounds to the French honeybee population, thanks to historical or ongoing trade between beekeepers. A total of 1513 colonies were sampled and sequenced: 1189 from France, 185 from Switzerland, 37 from Luxembourg, 37 from the Netherlands, 15 from Sweden and 50 from New Zealand. Out of these 1441 colonies were also phenotyped, after satisfying the condition that each beekeeper contributed at least 6 colonies (from 6 to 125 with on average 19.25 colonies). These colonies were phenotyped for multiple traits known to be related to varroa resistance (described in detail below), once per colony at the end of the beekeeping season (summer and autumn, i.e., typically between July and September in Europe).

### 2.2 Genetic Characterisation of Honeybee Colonies

All colonies were genotyped using the pooling and whole genome sequencing strategy described in Eynard et al. (2022). This study developed a strategy for characterising the average colony genetic composition, taking in account the specificities of honeybee genetics and colony social organisation, by sequencing large pools of honeybee workers, which can represent an average of the genetics of the colony and are also the individuals responsible for resistance trait performance within colonies. Briefly, three steps were involved for each colony: first allele counts at selected single nucleotide polymorphisms (SNPs) were obtained from the whole genome sequence, then the genetic background of the colony was estimated from a subset of discriminating markers, and finally the genotype of the queen was predicted among colonies of similar genetic background. These three steps are detailed below.

#### 2.2.1 Whole Genome Sequencing

For each colony, approximately 500 honeybee workers were ground in 100 mL of TNE buffer. Then, 15 mL of ground sample was then collected and centrifuged for 15 min at 3400 rcf (relative centrifugal force). A volume of 200 μL of supernatant was lysed overnight at 56°C, with a solution of proteinase K (Eurobio GEXPRK01‐B5) and DTT (1,4 dithiothreitol). Automated DNA extraction was done with a Qiasymphony‐Qiagen. DNA concentrations for each sample were estimated with Infinit200‐Tecan. Thereafter, pool sequencing was done on a NovaSeq6000 platform in order to obtain about 30X genome wide raw sequencing coverage. Sequencing reads were aligned to the honeybee reference genome Amel HAv3.1 (Genbank accession GCA_003254395.2; Wallberg et al. 2019), using BWA‐MEM (Li 2013). In addition, it was expected that the biological sample contained adult varroa, present in the phoretic phase on the bees, within the pool of sequenced honeybee workers. The obtained reads were therefore also aligned to the varroa mitochondrial genome from the Vdes_3.0 assembly (Genbank accession GCA_002443255.1; Techer et al. 2019) using the same procedure.

#### 2.2.2 SNP Genotyping

Genotypes were estimated at each of the 7,023,976 high‐quality SNPs identified in Wragg et al. (2022). Pool sequences were analysed using Samtools mpileup (Li and Durbin 2009) with the recommended parameters: –C minimum mapping quality for reads with excessive mismatches of 50, −q minimum mapping quality for an alignment of 20, −Q minimum base quality of 20. Then, Pileup files were interpreted by the PoPoolation2 utility mpileup2sync (Kofler, Pandey, and Schlötterer 2011), with a minimum quality of 20 and were finally converted to allele counts and sequencing depth files, filtering out real tri‐allelic and potential sequencing error. This procedure led to a set of 6,831,074 SNPs that were used in all downstream analyses. Colonies were sequenced on average at 27.4X coverage and each selected SNP at 29.9X coverage, as planned by our study design (Table S1).

#### 2.2.3 Population Structure

For each colony, we ran the model presented in Eynard et al. (2022) to estimate the genetic background on 48,589 SNPs selected for their capacity of differentiating the three main European genetic backgrounds (Wragg et al. 2022; Dogantzis et al. 2021; Obšteter et al. 2022): the *C* lineage, comprising the mildly differentiated *Apis mellifera ligustica* and *Apis mellifera carnica* ; the *M* lineage of Western Europe *Apis mellifera mellifera* and the *O* lineage of Eastern Europe/South‐Western Asia *Apis mellifera caucasia*. Specifically, the SNPs were chosen based on the following criteria: (i) a maximum of two polymorphic sites within a 100 base pair window, (ii) only one representative marker per linkage disequilibrium (LD) block with $R^{2}$ higher than 0.8, (iii) the variance between allele frequencies in the three main European genetic backgrounds is higher than zero, to allow for population identification and (iv) minor allele frequencies (MAF) within the selected markers follow a uniform distribution. The list of selected markers is provided in Table S2. For step (ii) above, LD was estimated by using the reference diversity panel from Wragg et al. (2022) using the plink software version 1.9 (Purcell and Chang 2015; Purcell et al. 2007) with options ‐r2 ‐ld‐window 100 ‐ld‐window‐kb 10 ‐ld‐window‐r2 0.8 and a unique marker was selected manually as the median point for each LD block. The admixture model from Eynard et al. (2022) was used to estimate the genetic background of each colony (i.e., the admixture proportions; Pritchard, Stephens, and Donnelly 2000 of each of the three genetic backgrounds).

#### 2.2.4 Queen Genotype Reconstruction

Following the procedure described in Eynard et al. (2022) we grouped the colonies in categories based on their genetic background (*A. m. ligustica & carnica*, *A. m. mellifera*, *A. m. caucasia* and hybrid), so as to obtain homogeneous populations. Colonies were grouped in one of the three first categories if they harboured more than 80% of the same genetic background. Colonies that could not be assigned to such a homogeneous background were assigned to the ‘hybrid’ group. The group of pure *A. m. caucasia* colonies was too small (*n* = 21) to be considered further in the study. Once homogeneous groups are defined it is possible to perform honeybee queen genotype inference using the homogeneous model described in Eynard et al. (2022). In short, the method is based on the likelihood of the queen genotype, written as

(1) $$x_{l}^{c}|d_{l}^{c},f_{l},g_{l}^{c}∼\text{Binomial}\left(\frac{f_{l}+g_{l}^{c}}{2},d_{l}^{c}\right)$$

where $g_{l}^{c}$ is the (unknown) queen genotype, $f_{l}$ is the unknown reference allele frequency in the population, $d_{l}^{c}$ and $x_{l}^{c}$ the sequencing depth and allele counts obtained from pool sequencing for locus l and colony c. By considering all colonies of the same genetic background jointly, $f_{l}$ can be estimated by maximum likelihood and the posterior probabilities of the three possible genotypes of the queen can be computed.

### 2.3 Varroa Resistance Phenotypes

#### 2.3.1 Varroa Infestation

Varroa infestation was quantified with four different measures: phoretic mite infestation (on adult bees using two different methods), brood infestation and total mite load.

*Phoretic mite infestation* (*v_pho*) was measured using the detergent method (Dietemann et al. 2012). In brief, a sample of approximately 300 adult honeybees was collected in each colony, on a frame containing uncapped brood. After weighing this sample, the number of mites falling as a consequence of washing with a detergent solution was counted, and the proportion of mites within the sample expressed as the number of varroa per 100 honeybees (assuming the weight of 1 single bee to be 140 mg). An alternative measure of phoretic varroa (*v_mito*) was obtained from the pool sequencing data by calculating the ratio of the number of reads mapping to the varroa mitochondrial genome on the number of reads mapping to the honeybee genome sequence.

*Brood infestation* (*v_brood*) was expressed as the proportion of varroa infesting brood cells in the colony. This proportion was estimated among 300 randomly sampled brood cells on a single frame containing capped broods aged 7–11 days post‐capping (P5–P8 stages).

*Total mite infestation* (*v_load*) was estimated by combining the phoretic and brood infestations:

(2) $$v_{\text{load}}=v_{\text{brood}}\times n_{\text{brood}}+v_{\mathrm{pho}}\times \frac{n_{\mathrm{bee}}}{100}$$

where the total number of brood cells ($n_{\text{brood}}$) and adult bees ($n_{\mathrm{bee}}$) in the colonies were estimated using the ColEval method (Hernandez et al. 2020).

#### 2.3.2 Mite Non Reproduction

MNR, originally known as SMR, was estimated as detailed in Mondet, Parejo, et al. (2020). In brief, this estimate infers varroa reproductive status for each brood cell infested by a single varroa foundress and provides a proportion of reproductive mites in the colony. It was estimated on about 300 brood cells (some also used to determine mite brood infestation) with the aim to reach at least 35 single mite infested cells.

#### 2.3.3 Recapping of Infested Cells

The uncapping and further recapping of varroa infested brood cells by adult honeybees is a behavioural trait that has been shown to be associated with varroa resistance (Oddie, Dahle, and Neumann 2018). It can be estimated by dissecting brood cells to detect the presence (non‐recapped cell) or absence (recapped cell) of the larval cocoon silk in the cell cap (Büchler 2017). This was measured on the colony at the same time as measuring MNR. This trait is expressed as the proportion of recapped cells among the infested cells.

All phenotypes were recorded by technicians having followed an extensive training period prior to sampling. Moreover, for statistical analysis they were corrected to fit the assumption of Normality underlying genome wide association study (GWAS) models. Details on the transformations can be found in Methods S1.

#### 2.3.4 Phenotypic Characterisation of Colonies

The correlation between varroa‐associated phenotypes within and across groups were estimated using the traditional Pearson's method. A Principal Component Analysis (PCA) was performed using the R package FactoMineR (Lê, Josse, and Husson 2008). Due to scoring difficulties in the field some phenotypes are missing for some colonies (number of missing records for *v_pho n* = 106, *v_mito n* = 209, *v_brood n* = 112, *v_load n* = 165, *MNR n* = 112, *recap n* = 112 out of 1423 phenotyped colonies, after exclusion of the *A. m. caucasia* colonies). Imputation of these missing records was performed prior to analysis using the R package MissMDA (Josse and Husson 2016). Loadings of colonies on the first principal components (PCs) were used as a synthetic varroa infestation phenotype for genetic association tests.

### 2.4 Genome Wide Association Studies and Meta‐Analyses

#### 2.4.1 Genomic Relationship Matrix

For each genetic background identified, *A. m. ligustica & carnica*, *A. m. mellifera* and the hybrids, only SNPs with a MAF above 0.01 and missing rate below 5% were retained. A genomic relationship matrix (GRM) between colonies within each group was estimated on pool sequencing allele frequencies taking SNP LD into account through the SNP weights produced by LDAK (Speed et al. 2017; see Methods S2 for more details). Additionally, in order to further describe the genetic structure within each group, a PCA on the GRM was performed, using LDAK (Speed et al. 2012). Horn's parallel analysis (Dinno 2009) was used to decide on the number of PCs to keep as covariates in the linear model for explaining the variance. Twenty, 12 and 16 first components were retained from this PCA for *A. m. ligustica & carnica*, *A. m. mellifera* and the hybrid colonies respectively.

#### 2.4.2 Genome Wide Association

Genome wide association study were performed for three traits: varroa infestation (here‐after called *varroa_inf*), *MNR* and recapping of varroa infested cells (*recap*). Each GWAS tested the association between the reconstructed queen genotypes and the phenotype using the univariate linear mixed model (lmm) as proposed in GEMMA (Zhou and Stephens 2012) at each SNP in turn (LMM‐GWA), resulting for each SNP in an estimate of its effect and associated standard error, as well as a *p* value. Association studies were performed for all markers initially available, after filtering for MAF above 0.01 and missing rate below 5% (3,084,335 reconstructed queen's genotypes for the *A. m. ligustica & carnica*; 2,729,072 for the *A. m. mellifera* and 3,185,994 for the hybrid individuals respectively). Polygenic effects were accounted for with the GRM described above. In addition, further correction was performed by adding as covariates the PCs from the PCA of the GRM, selected as explained above. This was done to correct for the effects of unmeasured confounders with the genetic structure on the phenotypic variation (such as apiaries, beekeeper, year etc. effects). In Methods S2, we illustrate how the structures of the GRMs correlate somewhat to different environmental structures in the data.

To assess the effectiveness of the correction for population structure, the genomic inflation factor $\lambda_{\mathrm{gc}}$ was estimated as the median of the chi‐squared test statistics divided by the expected median of the chi‐squared distribution under the null hypothesis. Parameter $\lambda_{\mathrm{gc}}$ ranged between 1.02 and 1.08 for the GWAS on *A. m. ligustica & carnica*, between 0.98 and 1.03 for the GWAS on *A. m. mellifera* and between 0.99 and 1.04 for the GWAS on the hybrid colonies therefore showing essentially no inflation or deflation of the *p* values associated with the tested SNPs (Figure S1).

To determine the significance of each SNP for a trait, a false discovery rate procedure was applied, using the adaptive shrinkage method (Stephens 2017) as implemented in the ashr R package. Specifically, SNPs with a local false discovery rate (lfdr) and local false sign rate (lfsr) lower than 0.1 were deemed significant. The lfdr is the probability, knowing the observed data, that an effect is erroneously declared significant and lfsr is the probability, knowing the observed data, that the sign of an effect declared significant is wrong (Stephens 2017). The proportion of phenotypic variance explained by the SNPs (PVE), and its standard error, was estimated by the univariate linear model (LMM‐GWA) and using the Bayesian sparse linear mixed model (bslmm, BSLMM‐GWA), with default gamma parameters of 0–300 SNPs, 1,000,000 sampling steps and 100,000 burn‐in iterations, as proposed by GEMMA (Zhou, Carbonetto, and Stephens 2013). This model was fitted with the GRM and associated covariates, exclusively for LMM‐GWA, and we estimated the proportion of genetic variance explained by the sparse effects (PGE) of the trait as well as 95% credible intervals from empirical estimates.

#### 2.4.3 Meta‐Analysis

Genome wide association study results for the same phenotype on the three ‘pure’ genetic backgrounds were combined with two meta GWAS methods: (i) MANTRA (Morris 2011) a meta‐analysis method dedicated to combine GWAS results from different genetic ancestries, and (ii) mash (Urbut et al. 2019) a general purpose data‐driven Bayesian meta‐analysis method modelling SNP effects with a mixture of multivariate Gaussian distributions with different correlation matrices.

MANTRA and mash were run on all SNPs, using effects (β) and associated standard errors estimated with LMM‐GWA of GEMMA. For mash inferences, canonical and data‐driven covariance matrices were used. The canonical matrices were estimated automatically by mash. Data‐driven matrices were: (i) estimated based on extreme deconvolution from PCA matrices, (ii) based on $F_{s}t$ values between populations, similar to MANTRA and (iii) based on correlation between SNP or gene effects in the different groups. Mash includes an estimation of the residual correlation. In this analysis, the simple residual correlation estimation model was preferred, as it outperformed more complex residual correlation estimation models. SNPs with a log10(Bayes Factor) > 5 with MANTRA were called significant, a threshold which was shown to be conservative by Wang et al. (2013). Mash automatically assigns significance to each marker. In our study the corresponding log10(BF) threshold varied from 1.16 to 1.4 depending on the trait.

Genetic correlations were estimated using Pearson's correlation coefficients on the allele effects for each SNP, calculated by our different GWAS methods (individually with GEMMA and ash, and across co‐ancestries with MANTRA and mash).

#### 2.4.4 Gene Prioritisation

The variant effect predictor (VEP) tool from Ensembl (McLaren et al. 2016) was used to identify the impact of each variant of each significant SNP on the corresponding honeybee genome annotation (stop codon, gained or lost, missense, frameshift…), its closest genes and their locations (upstream, downstream, intronic or exonic region…). Additionally, we identified distant genes, in conserved haplotype regions, containing variants in high LD ($R^{2}>0.8$) with the significant SNP. Linkage disequilibrium was computed, for each group, using data from the genetic diversity reference panel Wragg et al. (2022) for each significant SNP with all other variants on the same chromosome using the plink software version 1.9 (Purcell and Chang 2015; Purcell et al. 2007) with options ‐ld‐window‐r2 0.8 ‐r2 inter‐chr.

In addition, it is crucial to integrate all existing studies in order to have the best a priori knowledge of varroa resistance mechanisms. Therefore, to confront our results with previous work, we took advantage of the recent review by Mondet, Beaurepaire, et al. (2020). Compared to the studies reported in this review, our analysis tackles a much larger genetic diversity of honeybee populations in Europe, as well as beekeeping practices representative of the diversity of colony management strategies. To compare genome regions detected across studies, we transformed genome coordinates for the previous studies described in Mondet, Beaurepaire, et al. (2020) into coordinates for the latest genome assembly (HAv3.1; Wallberg et al. 2019) such as used here, by performing a liftover (Table S3).

## 3 Results

### 3.1 Genetic and Phenotypic Diversity of Honeybee Colonies

**FIGURE 2:**
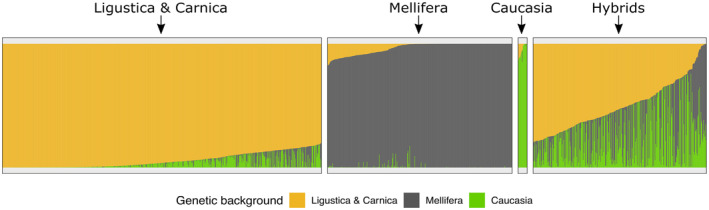
Genetic backgrounds for each colony, per group. Proportion of genetic backgrounds from subspecies in four groups of colonies. Vertical bars represent colonies with subspecies proportions. Yellow: *A. m. ligustica* or *A. m. carnica*; black: *A. m. mellifera*; green: *A. m. caucasia*. The three larger groups: *A. m. ligustica & carnica, mellifera* and hybrids were selected for GWAS.

Using allele frequencies estimated from pool sequence data for the 1513 sampled honeybee colonies, we identified three main ancestry groups: 703 colonies were identified as having more than 80% *A. m. ligustica & carnica* genetic background, 407 as having more than 80% *A. m. mellifera* genetic background and 382 as hybrids (Figure 2). An additional 21 colonies were found to be of pure *A. m. caucasia* ancestry, but due to the small sample size of this category they were not analysed further.

**FIGURE 3:**
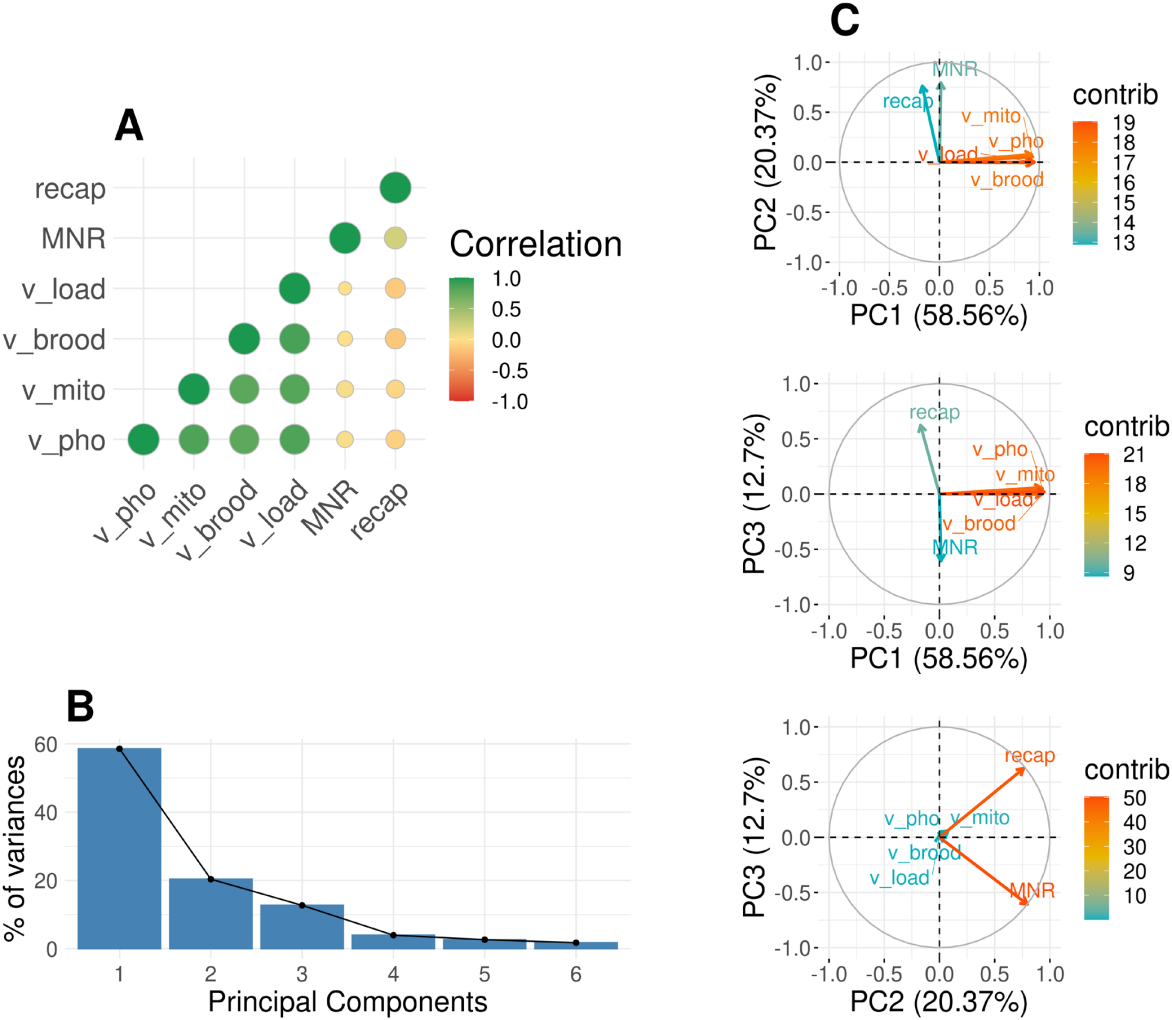
Correlation and principal component analysis. Description of the correlation between phenotypes and PCA. Panel (A) shows the correlations between our original phenotypes, with larger dots for stronger, green for positive and red for negative correlations. Panel (B) summarises the percentage of variance explained by each of the PCs from axis 1 to 5. Panel (C) shows our phenotypes on PCA for axis 1, 2 and 3, the colour gives the contribution of each variable to the axis, with red as the highest. The correlation and PCA estimates are based on an analysis of the dataset including colonies from the three groups *A. m. ligustica & carnica*, *A. m. mellifera* and the hybrids (*n* = 1423).

For the purpose of this study, we collected six phenotypes on these colonies. Out of the six phenotypes initially available, four were associated with varroa infestation (on the adult bees: phoretic infestation rate *v_pho* and varroa mitochondrial sequence reads *v_mito*, inside the brood: brood infestation rate *v_brood* and overall in the colony: varroa load *v_load*). These were highly positively correlated with each other (R between 0.79 for the correlation between *v_pho* and *v_brood* and 0.88 for the correlation between *v_brood* and *v_load*), *p* values $<10^{-16}$ and drove the first dimension of the PCA across all colonies (Figure 3), and also within each group (Figure S1), with about 60% of the variance explained in each case. The first component of the PCA was used as a synthetic varroa infestation phenotype thereafter (*varroa_inf*). The two remaining phenotypes were expected to be linked to resistance to varroa infestation, either through mechanisms repressing varroa reproduction, and thus varroa population growth within the colony (*MNR*), or through the cleaning of brood cells infested by varroa (*recap*). *MNR* was slightly positively correlated (*R* = 0.23, *p* value $<10^{-16}$) with the recapping of infested brood cells, as expected under the assumption that *recap* contributes to overall *MNR*. *MNR* was not correlated with varroa infestation (*R* close to and non‐significantly different from zero). *recap* was slightly negatively correlated with varroa infestation (*R* between −0.15 for the correlation between *recap* and *v_brood* and −0.07 for the correlation between *recap* and *v_mito*, *p* values < 0.0045). This observation is consistent, as *recap* is one of the mechanisms linked to a reduction in varroa infestation within the colony. *MNR* and *recap* both contributed to the second dimension of the PCA, explaining about 20% of the variance. They are separated on the third axis of the PCA, which explains about 12% of the variance (Figure 3). GWAS were performed on each of these three phenotypes (*varroa_inf*, *MNR* and *recap*).

### 3.2 Meta‐Analysis of Varroa Resistance

Our aim with the meta‐analysis was to identify markers significantly associated with our traits of interest across the three main honeybee genetic backgrounds found in Europe. Within populations, we used a standard linear mixed model approach, implemented in GEMMA (Zhou and Stephens 2012). Significant genetic markers (SNPs) were identified relative to their local false discovery and false sign rates, estimated by adaptive shrinkage (Stephens 2017). Population level analyses were combined into a meta‐analysis using two Bayesian methods: a co‐ancestry specific program MANTRA (Morris 2011) and the more generalist mash (Urbut et al. 2019), to increase power to detect significant markers across the three genetic backgrounds.

#### 3.2.1 Associated Variants

The individual GWAS for each genetic background group and each phenotype allowed us to identify 8 genomic regions of interest (nine SNPs). A detailed list of the SNPs can be found in Table S1.

In detail:

- For *varroa_inf*, we found one variant (1:10080627:C > T) in *A. m. ligustica & carnica*, with a positive alternative allele effect, and one (4:11665460:G > A) in the hybrid group, with a negative alternative allele effect.
- No significant variants were identified for *MNR*.
- For *recap*, we identified four significant variants in *A. m. mellifera*. For two SNPs, the alternative alleles had negative effects on the trait (2:2729874:T > C and 3:1059430:T > C), whereas the two others had positive effects (4:4327611:G > A, 15:8485332:G > A). In *A. m. ligustica & carnica*, a region with two SNPs was found, for both of which the alternative alleles had positive effects on the trait (2:12025610:A > G, 2:12025647:A > G). Finally in the hybrid group, one variant was detected for which the alternative allele had a negative effect on the trait (13:9483955:C > T).

Out of these nine significant SNPs, eight were located inside genes described in the honeybee annotation (Wallberg et al. 2019) and one was located 7 kb upstream of the closest gene (for more details see Table S1).

Across the three traits, the meta‐analysis allowed the identification of 51 genomic regions containing 56 significant SNPs: 14 (*n* SNPs = 14) were significant for *varroa_inf*, 14 (*n* SNPs = 15) for *MNR* and 23 (*n* SNPs = 27) for *recap*. These regions were distributed across the whole genome, involving almost every chromosome (for a detailed list of the SNPs, see Table S2).

In detail:

- For the *varroa_inf* trait: Of the 14 SNPs, four were located on chromosome 7; two each on chromosomes 1, 5 and 8 and one on chromosomes 4, 6, 11 and 12. Thirteen of these SNPs were considered significant in the MANTRA meta‐analysis with log10(BF) ranging from 7.59 to 5.09 and one was significant in the mash analysis with log10(BF) = 1.16. For six of these variants, the alternative allele had a positive effect on the trait in at least one of the groups (5:9190579:A > G, 7:5772089:A > T, 7:6738985:T > A, 7:11806658:G > A, 8:9799408:C > T and 12:10734707:A > G), for seven alternative allele had a negative effect on the trait, in at least one of the groups (1:20960056:C > T, 1:25184394:C > T, 4:11665460:G > A, 5:75369:C > T, 6:10450971:C > T, 7:5762037:T > C and 8:2468335:C > T) and for one the sign of the effect depended on the group (11:9369229:T > C). Eight SNPs were found inside genes from the honeybee annotation, six in introns, one in a 5’ UTR and one in a 3’ UTR region. Out of the six remaining SNPs, three were within 11 kb upstream and three 54 kb downstream the closest gene.
- For *MNR*: of the 15 SNPs, four were located on chromosome 1; two each on chromosomes 8, 10 and 12 and one each on chromosome 2, 3, 5, 11 and 15. All these SNPs were considered significant in the MANTRA meta‐analysis with log10(BF) ranging from 5.12 to 6.44. For three of these variants, the alternative allele had a positive effect on the trait, in at least one of the groups (2:4437645:G > A, 12:10153855:A > G and 15:4853529:C > T), for seven it had a negative effect on the trait, in at least one of the groups (1:16327085:C > T, 1:21374478:G > A, 1:24201224:C > T, 3:6206342:C > T, 8:1150346:C > T, 11:9527267:G > A and 12:136634:G > C) and for five the sign of the effect depended on the groups (1:2891204:G > A, 5:2008472:A > C, 8:9557205:C > T, 10:5359169:T > A and 10:5359173:C > T). Out of the 15 significant SNPs, 12 were found inside genes: 10 in introns, one in a 3’UTR region and one causing a missense variation. The three remaining SNPs were within 17 kb downstream the closest gene.
- Finally, for *recap*: of the 27 SNPs, four each were located on the chromosomes 2 and 7; three each on the chromosomes 1, 5 and 14; two each on chromosomes 4 and 15 and one each on chromosome 3, 8, 9, 10, 11 and 16. Of these SNPs, 26 were considered significant based on their log10(BF) values for the MANTRA meta‐analysis with log10(BF) ranging from 5.01 to 7.61 and six had log10(BF) values higher than one from the mash analysis, with log10(BF) ranging between 1.41 and 2.53. Five SNPs for *recap* were found significant in both the MANTRA and mash meta‐analysis, while for the other two traits, no SNPs were found significant in both analyses. For these five SNPs, the log10(BF_MANTRA) ranged between 5.03 and 7.61 and the log10(BF_mash) between 1.41 and 2.53. These SNPs were located on the chromosomes 2 (two SNPs), 3, 11 and 15. For eight of the SNPs, the alternative allele had a positive effect on the trait, in at least one of the groups (1:7448807:A > T, 1:7448811:T > C, 4:7321246:T > A, 4:7321247:G > T, 14:6686131:A > G, 14:8481541:A > G, 15:2081876:A > G and 15:8485332:G > A), for 18 SNPs the alternative allele had a negative effect on the trait, in at least one of the groups (1:15280956:G > A, 2:2729874:T > C, 2:8350714:G > A, 2:12025610:A > G, 2:16060868:G > A, 3:1059430:T > C, 5:6736534:T > C, 5:6761414:T > A, 5:8737386:G > A, 7:7028040:G > A, 7:7051965:A > G, 7:7078376:C > T, 8:1551638:C > T, 9:11564671:A > C, 10:2026877:C > G, 11:14369154:G > C, 14:3782741:G > A and 16:1812909:C > T) and for one SNP the effect depended on the genetic group (7:8466948:A > G). Out of the 27 significant SNPs, 18 were found inside genes: 16 in introns, one in a region coding for long non coding RNA and one in a 5’UTR, being also a missense variant. Three SNPs were found between 52 and 102 kb downstream of the closest genes. The remaining six SNPs were found between 5 and 129 kb upstream of the closest genes (for details, see Tables S2 and S3).

**FIGURE 4:**
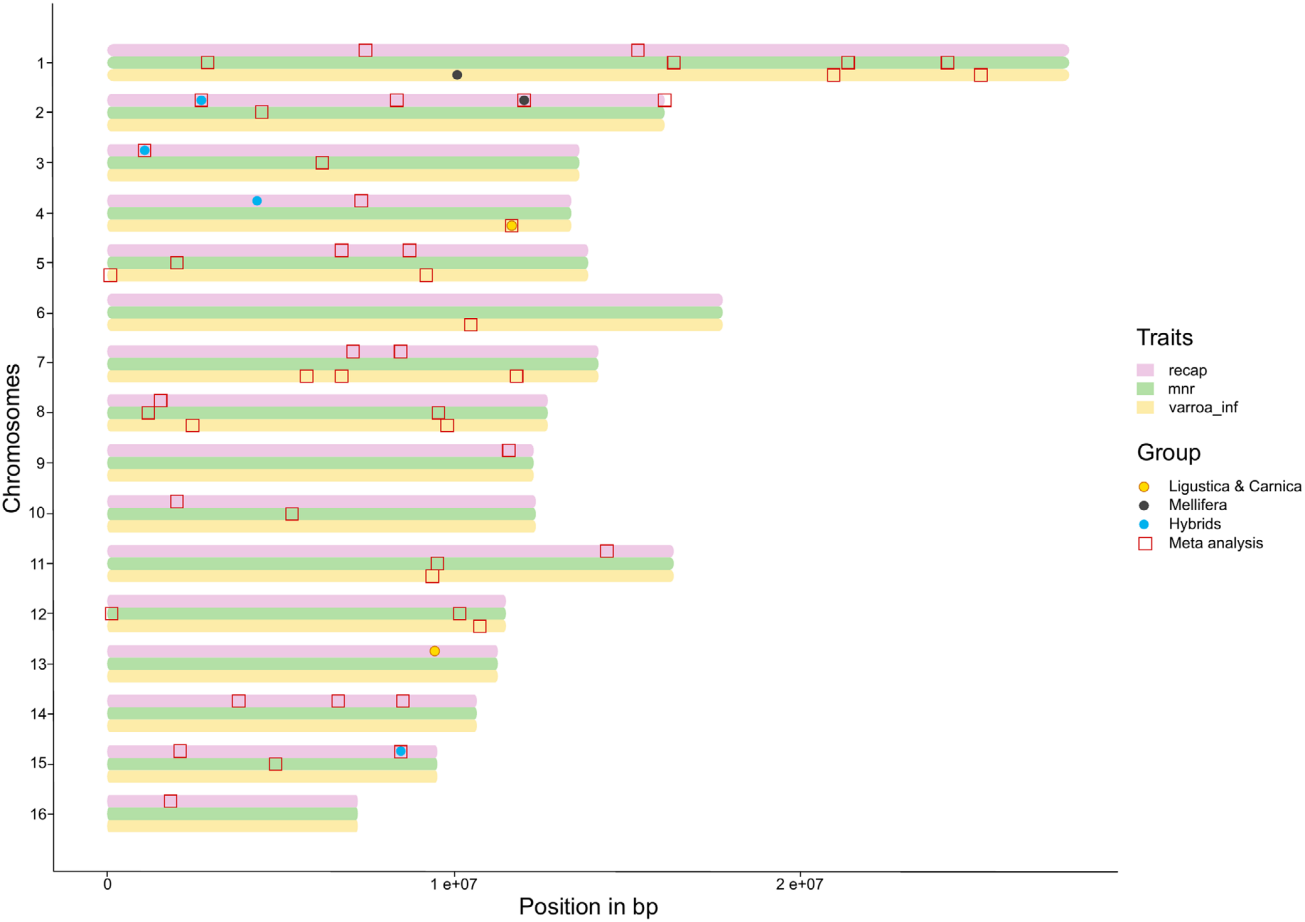
Overlap between traits across significant regions. Position of each of the significant SNPs on the honeybee chromosomes: a coloured dot means that this position has been identified either in *A. m. ligustica & carnica*, in *A. m. mellifera* or in hybrids in individual GWAS; a red square means that it has been identified in the meta analysis. The coloured bars represent the phenotypes of interest *varroa_inf* (yellow), *MNR* (green) and *recap* (pink). This figure allows us to see overlapping windows containing significant markers across the phenotypes.

Next we identified chromosome regions smaller than 1 Mb that shared significant SNPs between multiple traits. Between *varroa_inf* and *MNR* we identified a total of five such overlapping regions (*p* value = 6.4 × 10^−4^). Two were on chromosome 1 in the regions 20.9–21.4 and 24.2–25.2 Mb, one was on chromosome 8:9.5–9.8 Mb, one on chromosome 11:9.3–9.6 Mb and one on chromosome 12:10.1–10.8 Mb. Between *varroa_inf* and *recap* we identified a region on chromosome 5:8.7–9.2 Mb, one region on chromosome 7:6.7–7.1 Mb and one region on chromosome 8:1.5–2.5 Mb (*p* value for these overlaps = 0.042). Finally between *MNR* and *recap* we identified one region on chromosome 8:1.1–1.6 Mb. Statistically the overlaps between *varroa_inf* and *MNR* were highly significant, and between *varroa_inf* and *recap* slightly significant, assuring us that the marker co‐occurrence was non‐random (Table 1). (Figure 4, for the details of each region see Table S4).

**TABLE 1:** Overlapping regions. Number of overlapping regions between each trait and *p* value associated with the probability of these associations being non‐random.

	*Varroa_inf* (*n* regions = 14)	*MNR* (*n* regions = 14)
*MNR* (*n* regions = 14)	5 (6.4 × 10^−4^)	
*Recap* (*n* regions = 23)	3 (0.042)	1 (0.79)

#### 3.2.2 Heterogeneity of Effects

Three groups of colonies were analysed, based on genetic homogeneity. Two have relatively pure genetic backgrounds, corresponding to the two main lines of honeybee subspecies in Western Europe, and the third consists of varying degrees of hybridisation between pure groups. The $F_{\mathrm{st}}$ (a measure of genetic differentiation between groups) between groups was 0.26 between *A. m. mellifera* and *A. m. ligustica & carnica*, 0.20 between *A. m. mellifera* and hybrids, and 0.08 between *A. m. ligustica & carnica* and hybrids.

As detailed earlier, we observed 17 SNPs in the meta‐analysis (six for *varroa_inf*, three for *MNR* and eight for *recap*) for which alternative allele had significant positive effects for at least one of the groups. For 32 SNPs (seven in *varroa_inf*, seven in *MNR* and 18 in *recap*) we saw significant negative effects for at least one of the groups. Finally for seven SNPs (one in *varroa_inf*, five in *MNR* and one in *recap*) we observed a divergent effect depending on the group (Table S3). We noticed that marker effects estimated with GEMMA lacked precision, whereas the ash and mash methods applied stronger shrinkage to the estimates bringing most of them closer to zero. Going from individual analysis to meta‐GWAS improved the power to detect associations and improved our ability to accurately estimate the markers' effects.

Around 80% of the significant SNPs identified were located in intronic regions, and there were no differences between the annotations of the significant SNPs on the genome and the annotations of all tested SNPs, that is, there was no enrichment for a specific genomic feature. Five SNPs were found to be significant in both the GWAS performed on the separate groups and in the meta‐analysis: one for *varroa_inf* significant in the hybrid group, three for *recap* in the *A. m. mellifera* group and one for *recap* in the *A. m. ligustica & carnica* group. All five SNPs were located within genes: *LOC102655235* and *LOC410853* on chromosome 2, *LOC409402* on chromosome 3, *LOC408787* on chromosome 4 and *LOC726948* on chromosome 15.

#### 3.2.3 Example of Associations

**FIGURE 5:**
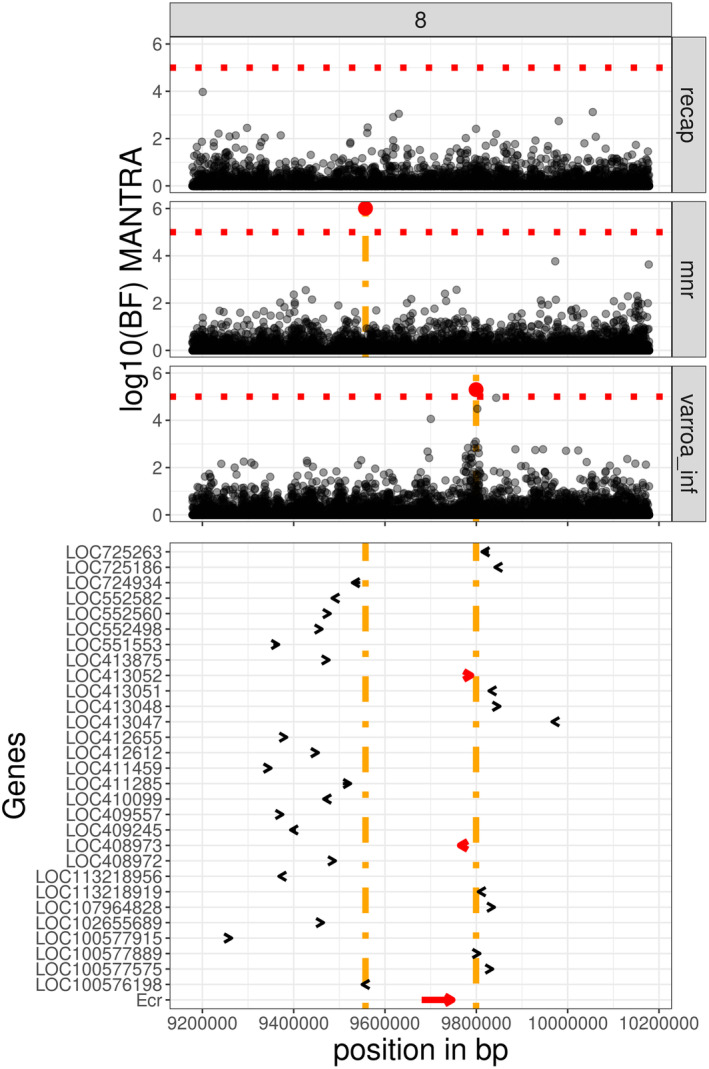
Significant region on chromosome 8:9.2–10.2 Mb. Results from GWAS on chromosome 8, region between 9.2 and 10.2 Mb, for *varroa_inf*, *MNR* and *recap*. Top panel: Log10 bayes factor for MANTRA for *recap* (top), *MNR* (middle) and *varroa_inf* (bottom). The red line indicates the significance threshold. Bottom panel: Genes from the honeybee annotation. The orange lines represent the positions of the significant markers, genes located between these markers are highlighted in red. The gene plotted at the bottom of the figure is our gene of interest ecdysone receptor *Ecr*.

One interesting region on chromosome 8 has two SNPs that were significant in the meta‐analysis: for *MNR* at 9,557,205 bp and for *varroa_inf* at 9,799,408 bp. These are in close vicinity to the ecdysone receptor (*Ecr*) gene (Figure 5). In addition, we also identified a SNP, located at 9,696,277 bp, within *Ecr*, in high LD with the significant SNP 8:2468335:C > T, that was also found in the meta‐analysis for *varroa_inf* (Table S5).

**FIGURE 6:**
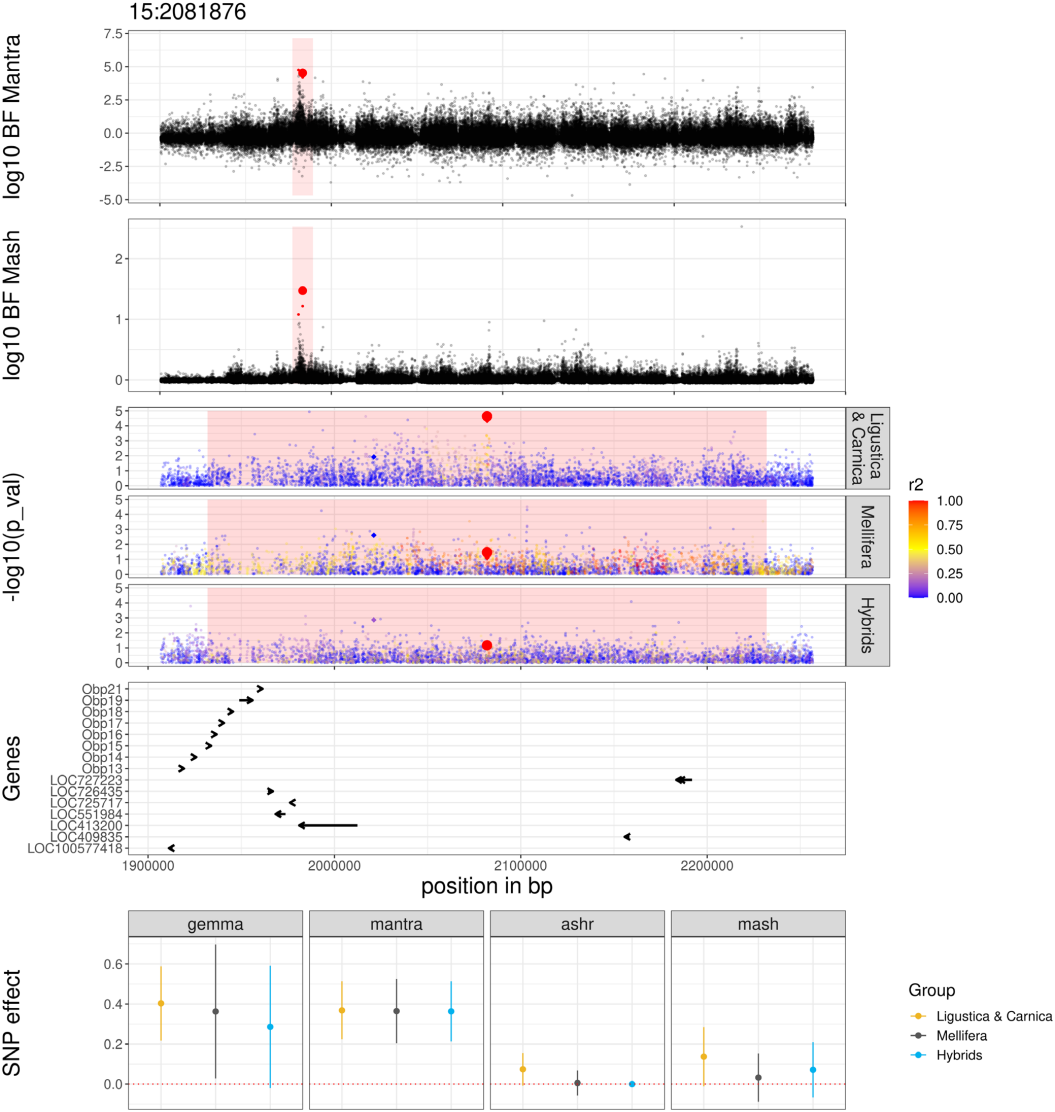
Significant region on chromosome 15 surrounding a significant marker. Plots show the region surrounding a significant marker positioned at 2,081,876 bp on chromosome 15. The significant marker is indicated by a large red dot and is centred in a 0.3 Mb region indicated as a pink background. Top two panels: Chromosome‐wise meta analyses with MANTRA and mash. Markers close to significance are indicated with smaller red dots. Middle three panels: −log10(*p* values), estimated for each of the three groups *A. m. ligustica & carnica*, *A. m. mellifera* and hybrids, with GEMMA. Markers are coloured according to their $R^{2}$ linkage disequilibrium with the focal marker. Genes panel: List and position of genes in the interval. Bottom: Effects of the significant marker estimated with GEMMA, ash, MANTRA and mash.

Another interesting region contains one SNP significant for *recap*, at 2,081,876 bp on chromosome 15 and two suggestive SNPs, close to the significant threshold, at positions 2,021,142 and 2,081,914 bp. The markers 15:2081876:A > G and 15:2081914:A > G were in full LD in *A. m. ligustica & carnica* and in the hybrid group and in high LD ($R^{2}$ > 0.8) in *A. m. mellifera*. They are located within the same 1.6 kb haplotype block identified by Wragg et al. (2022) (Table S6), which did not contain annotated genes in the current honeybee genome annotation. The marker 15:2021142:C > T is located in a short haplotype block (0.175 kb) overlapping the gene *LOC413200*, which has been identified as a putative immune related gene by Ryabov et al. (2014). Interestingly, they were located less than 1 Mbp downstream of a group of eight genes coding for odorant binding proteins (*Obp*). The alternative allele of the first SNP (15:2021142:C > T) had a negative effect for all groups, whereas that of the second (15:2081876:A > G) had a positive effect for all groups, particularly for *A. m. ligustica & carnica* (Figure 6).

### 3.3 Polygenic Architecture of Varroa Resistance

**FIGURE 7:**
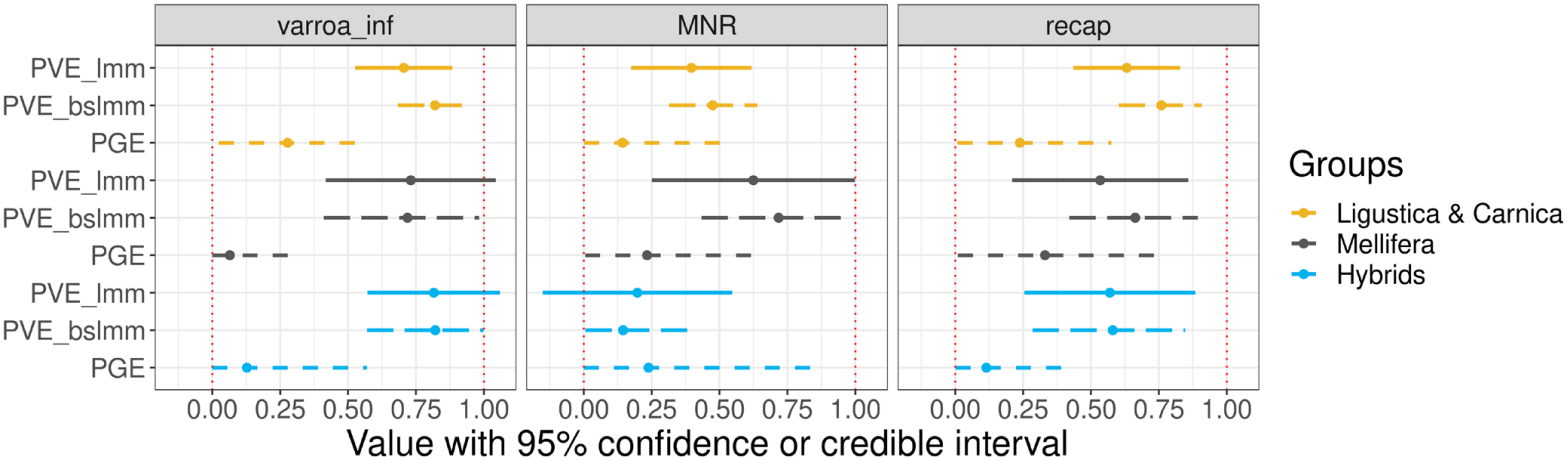
Genome wide association estimates. Confidence and credible intervals for the PVE by LMM‐GWA (full line) or BSLMM‐GWA (long dotted line) and the PGE explained by BSLMM‐GWA (short dotted line) for the three group analysed: In yellow *A. m. ligustica & carnica*, in dark grey for *A. m. mellifera* and in blue for hybrid and the three phenotypes *varroa_inf*, *MNR* and *recap*.

The Bayesian sparse linear mixed model (BSLMM‐GWA) (Zhou, Carbonetto, and Stephens 2013) implemented in the program GEMMA provides estimates for the proportion of PVE explained by the SNPs and PGE explained by the sparse effects, for each trait and in each group (Table 2 and Figure 7). PVEs estimated using BSLMM‐GWA ranged between 0.14 and 0.82 (se = [0.09; 0.19]) and were close to the LMM‐GWA estimates, which ranged between 0.20 and 0.82 (se = [0.07; 0.15]). The 95% confidence and credible intervals for PVEs from both LMM‐GWA and BSLMM‐GWA appeared exclusively positive, except for *MNR* in the hybrids group. However, PGEs were much lower, ranging between 0.06 and 0.33 (se = [0.08; 0.25]). Their 95% confidence/credible intervals often included zero. The only traits and groups having PGEs significantly different from zero were *varroa_inf* and *recap* for the *A. m. ligustica* group with PGEs of 0.277 and 0.238 respectively, and *MNR* for the *A. m. mellifera* group with a PGE of 0.23. The estimates for the GWAS on hybrids and *A. m. mellifera* always showed larger standard error, due to the smaller sample sizes of these groups. Interestingly, it appears that the PVE estimate is slightly higher for *A. m. mellifera* and the *MNR* phenotype compared to the two other groups (0.62 vs. 0.40 and 0.20), whereas they seem similar between the three groups for the two other phenotypes, *varroa_inf* (close to 0.75) and *recap* (close to 0.55) (a complete summary can be found in Table S7).

**TABLE 2:** Summary of phenotypic variance explained and proportion of genetic variance explained.

	Varroa infestation	Resistance to varroa infestation	
	*Varroa_inf*	*MNR*	*Recap*
**PVE(se) from LMM‐GWA**			
*Ligustica & Carnica*	0.705 (0.091)	0.396 (0.113)	0.632 (0.100)
*Mellifera*	0.731 (0.160)	0.625 (0.191)	0.534 (0.165)
*Hybrids*	0.816 (0.124)	0.197 (0.178)	0.570 (0.160)
**PVE[95% CI] from BSLMM‐GWA**			
*Ligustica & Carnica*	0.820 [0.683; 0.953]	0.475 [0.314; 0.640]	0.759 [0.602; 0.910]
*Mellifera*	0.719 [0.410; 0.982]	0.718 [0.433; 0.981]	0.663 [0.420; 0.893]
*Hybrids*	0.821 [0.570; 0.998]	0.145 [0.006; 0.393]	0.580 [0.285; 0.847]
**PGE[95% CI] from BSLMM‐GWA**			
*Ligustica & Carnica*	0.277 [0.024; 0.555]	0.143 [0.001; 0.503]	0.238 [0.008; 0.575]
*Mellifera*	0.065 [0; 0.286]	0.233 [0.005; 0.666]	0.331 [0.010; 0.751]
*Hybrids*	0.128 [0; 0.569]	0.238 [0; 0.880]	0.115 [0.001; 0.425]

Correlations between SNP effects across the whole genome, estimated using GEMMA and ash for individual GWAS analysis on the one hand, and MANTRA and mash for meta‐GWAS on the other, were mostly positive (Table 3, the detailed correlations can be found in Table S8). However, as expected there was almost no correlation between the different groups when estimated from individual GWAS (R ranged between 0.04 and 0.05 for *varroa_inf*, between −0.009 and 0.01 for *MNR* and between −0.005 and 0.02 for *recap*) whereas there was some positive correlation when estimated from meta‐GWAS (R ranged between 0.74 and 0.90 for *varroa_inf*, between 0.63 and 0.95 for *MNR* and between 0.34 and 0.89 for *recap*). Across the different phenotypes, *recap* and *MNR* appear to be slightly positively genetically correlated (R ranging between 0.02 and 0.20 depending on the association method and genetic group), as one could expect knowing that recapping behaviour, performed by adult bees, is potentially a component of the *MNR* phenotype. The phenotypes *varroa_inf* and *MNR* were not genetically correlated (R ranging between −0.02 and 0.07 depending on the association method and genetic group). Finally, *varroa_inf* and *recap* were mostly negatively correlated for *A. m. mellifera* and *A. m. ligustica & carnica* (*R* ranging between −0.30 and −0.02 depending on the association method and genetic group) whereas there was no to very little positive genetic correlation between these two phenotypes in the hybrids group (*R* ranging between 0.02 and 0.17 depending on the association method).

**TABLE 3:** Genetic correlations between traits and across methods and genetic groups. Range estimates of Pearson correlations between SNPs effects for our traits of interest calculated by different GWAS methods (GEMMA, ash, MANTRA and mash), on the different genetic groups.

		*Varroa_inf*	*MNR*	*Recap*
	***Varroa_inf***	1		
*Ligustica & Carnica*	*MNR*	[0.042; 0.049]	1	
*Mellifera*		[−0.023; 0.012]		
*Hybrids*		[0.050; 0.072]		
*Ligustica & Carnica*	*Recap*	[−0.304; −0.213]	[0.131; 0.176]	1
*Mellifera*		[−0.132; −0.020]	[0.088; 0.197]	
*Hybrids*		[0.021; 0.173]	[0.021; 0.119]	

## 4 Discussion

In this study, we performed the largest genome wide association study to date on the resistance of honeybees to their current biggest biotic threat, the parasite *Varroa destructor* . We combined an extensive genotyping and phenotyping effort with meta‐analysis methods to identify genetic markers and associated genes harbouring a significant effect on varroa resistance. We took advantage of having both multiple traits associated with varroa resistance and a complex genetic ancestry, characteristic of honeybee colonies. We were able to locate significant resistance effects to some regions of the honeybee genome (within specific genetic types and across the whole meta‐population) offering insights into the biological mechanisms involved in varroa resistance. The regions we identified in this study only explain a small portion of the genetic determinism for varroa resistance, meaning that the genetic variance of varroa resistance traits is highly polygenic. This study, based on about 1500 phenotyped honeybee colonies for which we sequenced pools of workers and genotyped around 3 million SNPs, benefits from the largest sample size (phenotyped and sequenced) known thus far and used for performing a GWAS in honeybees. This study is the most global association study for honeybee varroa resistance traits to date. The set of colonies included is highly representative of many honeybee populations worldwide, containing all the genetic backgrounds described in the diversity panel of Wragg et al. (2022). Our analysis stands out from the previous honeybee quantitative genetic studies that mostly focused on one specific genetic background, often in small experiments, not truly representative of the real‐world situation and for really specific phenotypes (see Guichard et al. 2021 for a review). In addition to the effort to gather the raw data, this study benefited from dedicated statistical methods for reconstructing honeybee queen genotypes from sequenced pools of workers (Eynard et al. 2022) and from meta‐analyses approaches used to increase statistical power to detect significant associations (Morris 2011; Urbut et al. 2019).

### 4.1 Phenotyping Varroa Resistance

Resistance to varroa is a complex trait, involving many different aspects of the biology of honeybee colonies. A recent extensive review of varroa resistance traits (Guichard et al. 2020) has revealed that there is no clear evidence for significant correlations between the standard traits measured as proxies for varroa resistance, such as VSH, grooming, MNR, hygienic behaviour or recapping behaviour. Depending on the population on which the trait has been measured, in particular their evolutionary history (e.g., natural or artificial selection), the correlations between these traits range from ‘apparent link’ to ‘no link’.

Here, we observe a slightly positive phenotypic correlation between traits linked to varroa resistance, *MNR* and *recap* (overall or at the population level), and a small negative correlation between these two traits and *varroa_inf* (Figure 3 and Figure S1). This finding fits with the hypothesis that there is a panel of mechanisms allowing honeybees to resist varroa and that these mechanisms do not seem to be completely shared across honeybee subspecies.

An important experimental aspect that limits varroa resistance studies, including this one, is that most of the currently applied measures of varroa resistance are difficult to scale to a large number of samples. For example, they can involve tedious and potentially subjective scoring, induced or artificial varroa infestation, multiple measures in time, estimation of ratios and applying heuristic thresholds for minimum detection. Nevertheless, direct estimates of varroa infestation remain the simplest traits to score for quantifying varroa resistance. Indeed, a low varroa infestation can be explained by multiple phenomena either linked to the environment, to beekeeping practices, to varroa biology or to a property of the honeybee colony itself. In this study, in addition to the classical measures of varroa infestation, we proposed to estimate varroa infestation indirectly, using the ratio of reads mapped to the varroa mitochondrial DNA over those mapped to the honeybee genome (*varroa_mito*). We believe this new measure offers specific advantages for the study of varroa resistance: (i) there is a high correlation between this estimate and the phoretic varroa infestation (Methods S1), a trait that is more complex to measure, (ii) both the colony genome sequence and the varroa infestation information come from a unique biological sample and therefore share their time points, (iii) there is no potential bias due to the operator, (iv) no specific technical skills are needed and (v) it is comparable across studies. As for other varroa infestation phenotypes, this measure can be impacted by the environment or the beekeeping practices. The cost of sequencing the sample to obtain the trait off‐sets the labour costs incurred with physically phenotyped traits. However, using it as a phenotype for varroa infestation would facilitate the establishment of a large collection of standardised phenotypic records, necessary to be able to build up information through time, follow phenotypic progress in a surveyed populations, perform genetic meta‐analyses and potentially breed honeybee populations for varroa resistance.

#### 4.1.1 Insights into Biological Mechanisms Underlying Varroa Resistance

Varroa resistance mechanisms can be partitioned into two types of traits. The first type includes traits related to hygiene (including VSH, recapping and MNR, but also more broadly grooming behaviour), involving first the accurate detection of varroa infested cells by worker bees, followed by their subsequent inspection/destruction. It has been shown that bees expressing the VSH behaviour (VSH bees) target more specifically than other bees cells with highly compromised brood, which is related to the level of infestation in the cells (Harbo and Harris 2005; Mondet et al. 2015). As a result, cells with fewer mites or mites that are not effectively reproducing are more likely to stay intact, thus increasing the level of MNR in the colony. The second type of traits are expressed by either the workers or the brood and disrupt the reproduction of the mites within capped cells (and thus increase MNR). Both trait types can reduce mite infestation in the colony, thus increasing varroa resistance of honeybee colonies. Interestingly, in this study we found genetic markers associated with genes that relate to these two categories.

##### 4.1.1.1 Impairment of Mite Reproduction

Two of our significant SNPs for *recap* were in a region around 7 Mb on chromosome 1 described as a potential quantitative trait locus (QTL) for VSH, in an association study by Tsuruda et al. (2012). Two other significant markers 1:2891204:G > A and 1:21374478:G > A were inside genes *LOC410758* and *LOC413968* respectively. These genes have been identified in a study by Saelao et al. (2020) when looking for selection signals in hygienic honeybee populations in the USA. In addition, our marker 11:14369154:G > C, which is significant for *recap*, is located close to the *TpnCi* gene, coding for troponin C type. This gene has been shown to be over‐expressed in non‐hygienic Africanised bee lines, when compared with hygienic lines, by Teixeira et al. (2021). Finally markers 4:10789077:T > C and 8:1551638:C > T were located in genes also found by Ament et al. (2011) and are believed to be involved in protein abundance in the fat body and haemolymph of the adult honeybee. We know that varroa, while infesting the colony, survives by feeding on these bee biological fluids: the fat bodies when on adult bees and the haemolymph when on pupae (Han et al. 2024). These markers might signal either an impact of varroa infestation, or the resilience to such infestation, on biological pathways linked to the composition of the honeybee haemolymph and fat body.

In the region on chromosome 8, we identified multiple significant markers close to the gene *Ecr*, ecdysone receptor. This gene, active in the vitellogenin production pathway, has been described as crucial for the reproduction of *Varroa destructor* , despite being produced by the bee host (as well as other arthropods). Our results are thus consistent with the *Ecr* gene, and more generally the vitellogenin pathway, as key factors in the interaction between honeybees and varroa (Conlon et al. 2019; Feldlaufer and Hartfelder 1997; Aurori et al. 2021).

##### 4.1.1.2 Detection of Varroa Infested Cells by Honeybees

When looking more into the general biology of the honeybee, we found two markers, significant for *MNR* on chromosome 10, located in the *5‐HT2beta* gene. This gene is a serotonin receptor involved in olfactory pathways in a large number of insects (French et al. 2014; Verlinden, Vleugels, and Vanden Broeck 2015). Its role in resistance to infestation could be related to the processing of cues sent to the adult bee to trigger behavioural traits and confirms the importance of olfactory pathways. We also noticed that the significant markers 3:12973246:A > G and 12973248:A > G located in *LOC413503* (GenBank accession alias GB41230) also found by Mondet et al. (2015), as being differentially expressed in the antennae of honeybees expressing VSH behaviour.

In the region on chromosome 15, we identified multiple significant markers located less than 1 Mb downstream from a group of genes coding for odorant binding proteins (*Obp*). These genes are found in two major clusters on the honeybee genome, with seven genes on chromosome 9 and nine on chromosome 15 (a monophyletic group called C‐minus subfamily). In addition, two genes are located on chromosome 10 and one each on chromosome 2 and 12. One gene remains un‐annotated on the current honeybee reference genome, giving a total of 21 genes (Forêt and Maleszka 2006; Mam, Karpe, and Sowdhamini 2023). The cluster located on chromosome 15 contains eight genes (*Obp13* to *Obp21*), six of which have already been mentioned in genomic studies looking at varroa resistance related traits (*Obp14*, *Obp15*, *Obp16*, *Obp17*, *Obp18* and *Obp21*; Mondet, Beaurepaire, et al. 2020; Gebremedhn et al. 2023). In particular, *Obp14* has been identified as upregulated in two studies looking at gene and protein expression in VSH bees (Mondet et al. 2015; Jiang et al. 2016; Hu et al. 2016). *Obp18* has been identified by two proteomic studies looking at the VSH and hygienic behaviour traits (Jiang et al. 2016; Hu et al. 2016; Guarna et al. 2015; McAfee et al. 2018). Even though these genes did not contain significant SNPs in the dedicated analysis, we can still hypothesise that they might be involved in some resistance mechanism targeting varroa infestation as they play a major role in sensory pathways. These genes might be relevant for marker assisted selection, as suggested by Marta Guarna et al. (2017), who developed a tool for selection for hygienic behaviour in Canadian honeybees.

### 4.2 Genetic Architecture of Varroa Resistance

The review by Guichard et al. (2020) reported heritabilities for traits associated with varroa resistance ranging from close to 0 to up to 0.85, with large standard errors. More recently, Gabel et al. (2023) estimated the heritability of *MNR* to be close to 0.4, which is in the same range as our estimates, between 0.20 and 0.40. Most 95% confidence/credible intervals for heritability estimates found in the literature included zero, while those estimates that did not were mostly modest (< 0.2). In addition, repeatability estimates for these traits are low (e.g., Büchler et al. 2020; Eynard et al. 2020). More importantly, estimates based on different populations, for example, *A. m. mellifera* or *A. m. ligustica & carnica* reveal differences that could be explained by different genetic architectures involved in these traits.

The heritability estimates for the honeybee resistance traits studied here seem high compared to standard traits measured on livestock species, which could be due to the fact that we are unable to completely distinguish genetic from environmental stratification. When intending to estimate heritabilities in honeybees, one faces the challenge to integrate the potentially large impact of environmental variation, as part of the population structure is possibly associated with such variation, in addition to genetic variation. In this study, we aim to correct for such structure by thoroughly accounting for covariates, PCs of the GRM. To validate our approach, we computed correlations between genetic, geographic and environmental distances of our colonies with a Mantel test and found that environmental variables are slightly correlated with population structure, whereas their correlation with our phenotypes of interest is not significantly different from zero (Table S9). Environmental variables seemed therefore to have some impacts that we do not take into account in our analysis (Table S10), and that might have slightly affected our heritability estimates with an upward bias. Such environmental bias can be considered as confounding factors in our GWAS analysis. Their extent can be measured by comparing the GWAS results from single locus GWAS (LMM‐GWA) or multi‐loci GWAS (BSLMM‐GWA), because the former corrects for confounding factors using PCs of the GRM while the latter does not. Consistent with a small effect of phenotype/genotype confounding, we found the PVE estimates with BSLMM‐GWA to be usually larger than those obtained with LMM‐GWA (Figure 7 and Table 2), although the difference was always very small. Overall, we cannot rule out some inflation of PVE estimates due to remaining confounding effects, but it is not likely to affect our general conclusion on the polygenic background of the traits analysed.

The PGE explained by large effects, estimated with BSLMM‐GWA (Zhou and Stephens 2012), was generally low and included zero. These estimates support our hypothesis that varroa resistance traits are highly polygenic and not simply driven by a few markers with large effects. The traits linked to varroa infestation and to resistance seem to have a small yet significant component of genetic heritability, and thus can be passed on from one generation to the next through selection. This is consistent with the few examples of the efficiency of artificial selection for honeybee resistance to varroa infestation (Harbo and Harris 2005, 2009). Our results, obtained here in more diverse honeybee populations than most studies, imply that genetic selection (natural or artificial) has the potential to drive increased varroa resistance in other genetic and environmental contexts, a positive perspective for honeybee populations worldwide. However, and even though we identified genetic markers with significant effects, it is unlikely that major causal mutations, explaining a big part of the phenotypic variance, can contribute significantly to this adaptive response.

### 4.3 The Future of Genome Wide Association Studies in Honeybees

The honeybee genome displays several unique characteristics. First, it is known to experience a large number of recombination events, with an average recombination rate of 37 cM/Mb (Wallberg et al. 2019). Second, the effective population size (i.e., the number of actively reproducing individuals) of a local population is expected to be rather large, due to the polyandric nature of the natural honeybee mating system, with each queen mating with multiple males from many other colonies, thus avoiding the over‐representation of a specific reproductive individual or genetic line. Such particularities cause low LD (Methods S2), making it harder to identify candidate loci (QTLs) linked to specific traits, to use in selection.

In addition, the honeybee population exhibits a complex genetic diversity. We provide here a better understanding of the genetic architecture underlying varroa resistance in honeybees in general, and in the French population in particular. Many honeybee colonies are hybrids, having varying proportions of the three main *Apis mellifera* subspecies found in Europe, namely *ligustica & carnica*, *mellifera* and *caucasia*. In this study, we took advantage of this admixed population to identify genetic markers linked with our traits of interest within genetic types, in hybrids and across these populations, highlighting differences in significance and direction of the effects depending on the genetic type. Indeed, markers with opposing effects across the different genetic backgrounds, especially between *A. m. ligustica & carnica* and *A. m. mellifera* were identified. Knowledge of LD and the associated identification of local haplotypes for genomic regions of interest, combined with knowledge of the effects of individual SNPs for each subspecies, can increase our prediction accuracy for different traits. A better understanding of the local genetic background of the hybrid population could help predict the effects of specific SNPs. Studies focusing on hybrid colonies, with their diverse genetic background scattered throughout the genome, and comparing different genetic make‐ups, could be highly valuable to identify relevant genetic patterns. As an example, multiple genome regions were flagged with more than one significant marker for the trait *recap*, but evidence linking these regions to honeybee biology is lacking. These regions should therefore be earmarked as potential regions of interest for future studies geared towards improving honeybee genome annotation and understanding the underlying biological pathways.

### 4.4 Selection on Honeybee Resistance to Varroa

One practical perspective of our work could be to integrate the identified SNP variants identified into genomic selection programs aiming at breeding for resistant honeybee colonies. Genomic selection is commonly used in mainstream livestock species but its application in global honeybee breeding is thus far lacking. In some countries, such as Germany, the selective breeding programmes are restricted to specific and rather homogeneous bee subspecies, such as *A. m. carnica*, limiting the genetic diversity available for effective selection. Mating schemes for which mating stations or artificial insemination are used for large‐scale breeding programs, are recorded in dedicated databases containing data for the majority of the German *A. m. carnica* breeding population (Lorenz 2016; Hoppe et al. 2020). In this context, some studies (Du et al. 2022a, 2022b; Hoppe et al. 2022; Uzunov et al. 2022; Bernstein et al. 2023) described the statistical models and sampling strategies that can be successfully applied to implement genomic selection in honeybees. One limitation to the widespread use of these methods is that so far genomic selection has been proven successful when the focus is on a single honeybee subspecies, for example, *A. m. carnica*, and concentrates the efforts on phenotypes linked to production. In the context of the French honeybee population, as described in this study, the hybrid stock as well as the complex phenotypes of interest, make it less straightforward to apply genomic selection directly. Traits with highly polygenic inheritance are challenging for selection and we expect that the markers identified here will not be sufficient for marker‐assisted selection, even though the abundant estimated variances for the phenotypes still supports the possibility of selection, as it has been pursued in the USA (Harbo and Harris 1999).

In future studies, a primary focus should be put on increasing further the sample size, in terms of the number of phenotyped and genotyped colonies, to boost the precision, detection power and replication capacity of association studies. Robustness of selection decisions could benefit from deeper pedigree records (Bernstein et al. 2023), as well as access to a large number of individual queen genotypes (Bernstein et al. 2021), rather than reconstructed ones. However, accessing this information implies a larger experimental burden, potentially limiting sample sizes. Hence, finding the right balance to optimise statistical power needs further evaluation. In addition, there is a need for standardised biological samples in terms of genotype and phenotype. The genotypes could either come from using genome wide SNP panels (Bernstein et al. 2023; Jones et al. 2020) or from whole genome sequencing characterised using a genetic diversity panel (Wragg et al. 2022). Phenotype collection could be automated further and with reduced variability, such as proposed here with the sequence‐derived infestation measure (*varroa_mito*).

## 5 Conclusion

Today varroa is a global threat to honeybee populations worldwide and is not likely to disappear. Sustainable management of varroa will require adapting honeybee populations to this new risk. This will involve multifactorial actions and by revealing the significant heritability of resistance traits, our study highlights that genetics is likely to play a role in this endeavour. While we show that many varroa resistance traits have a genetic determinism across a large diversity of honeybee colonies, this genetic determinism is not simple: it is very unlikely that a few mutations can be found that would confer genetic resistance to varroa in honeybees. While this could be seen as negative, as it will involve complex genetic management strategies, it also has the positive consequence that polygenic adaptation to varroa is more likely to be sustained in time, avoiding simple bypassing by varroa as is often seen for simple genetic resistance mechanisms.

Beyond the importance of our study to honeybee genetics, we believe it illustrates the great contribution that genomics can bring to the understanding of populations and their adaptation, not only in domesticated but also in wild species. With decreasing sequencing costs and the development of statistical methods to optimise the information retrieved from genomic data, understanding the genetic determinants of population adaptation is becoming feasible in many biological systems. In the face of the current challenges affecting biodiversity in general such information can offer a key contribution to the conservation of many species.

## Author Contributions

A.V., F.M., L.G., Y.L.C., B.B., B.S., F.P., A.D.: conceptualisation. S.E.E., F.M., B.B., B.S., A.V., Y.L.C., B.D., M.G., M.N.: data curation. B.S., S.E.E., A.V., F.M., F.G.: methodology. S.E.E., B.S., F.M., A.V.: investigation. S.E.E., F.M., B.S., A.V.: visualisation. B.S., F.M., A.V., B.B., L.G., Y.L.C., A.D.: supervision. L.G.: project administration. F.M., L.G., A.V., Y.L.C., B.B., A.D., B.S., F.P.: funding acquisition. S.E.E., F.M., B.S., A.V.: writing original draft. L.G., B.B., A.D., Y.L.C., F.P., F.G., B.L., J.M., B.D., M.G., M.N., K.T., O.B.: writing review and editing. S.E.E., B.S., F.M., A.V., B.B., L.G., Y.L.C., A.D., F.P., F.G.: formal analysis. K.T., R.M., E.L., O.B., B.L., J.M., B.D., M.G., M.N.: resources.

## Conflicts of Interest

The authors declare no conflicts of interest.

## Supporting information


**Figure S1:** QQplot for the GWAS on each group and phenotype. QQplot representing the observed −log10(*p* values) as function of the expected −log10(*p* values) for each of the GWAS performed with GEMMA for the three groups *A. m. ligustica & carnica*, *A. m. mellifera* and hybrids, for the three phenotypes of interest, varroa infestation (*varroa_inf*), *MNR* and recapping (*recap*). Parameters $\lambda_{\mathrm{gc}}$ calculated for each of these analyses.
**Methods S1:** Details on phenotypes and statistical transformations applied for association study.
**Methods S2:** Details on the genomic relationship matrix estimations and description of the SNP weights estimated by LDAK.

**Table S1.** Detailed sequencing information for each colony. Total number of SNPs, number of SNPs not sequenced, number of SNPs sequenced, average sequencing depth, minimum sequencing depth, maximum sequencing depth, standard deviation of the sequencing depth.
**Table S2:** List of the 50 k SNPs used for genetic background determination.
**Table S3:** Liftover of the regions listed in the review by Mondet, Beaurepaire, et al. (2020) from the honeybee reference genome Amel4.5 to the current reference genome AmelHAV3.1. The informations from the review are: name, chromosome, start, end of the region strand, size, as well as the information after liftover to AmelHAV3.1: chromosome, start and end of the region, strand, size, coverage, type, the character of interest and associated literature reported in the review.
**Results S1:** List of the SNPs identified as significant for one of the phenotypes of interest in the GWAS per group. Details of the summary statistics for these SNPs are presented: phenotype of interest, group, chromosome, SNP name, position, region (locus), SNP (Reference/Alternative), alternative allele effect, standard error of the effect, *p* value, local false sign rate (lfsr), s‐value, local false discovery rate (lfdr), q‐value, posterior mean from ash, posterior standard deviation from ash, closest locus, alias name, variant type from VEP, locus start position, locus stop position, locus strand, SNP position to the closest locus, SNP distance to the closest locus, mention of this SNP/locus in literature, associated phenotype in literature.
**Results S2:** List of the SNPs identified as significant for one of the phenotypes of interest in the meta GWAS per group. Details of the summary statistics for these SNPs are presented: phenotype of interest, chromosome, SNP name, position, region (locus), SNP (Reference/Alternative), number of groups in which the SNP is present, log10 BF for MANTRA, posterior probability for MANTRA, number of tested samples, direction of the effect in the different groups, log10 BF for mash, number of significant signal in mash, closest locus, alias name, locus start position, locus stop position, locus strand, variant type from VEP, SNP position to the closest locus, SNP distance to the closest locus, mention of this SNP/locus in literature, associated phenotype in literature, comments.
**Results S3:** Effects of the alternative allele for each of the SNP identified as significant in one or more analyses. In detail: SNP name (chromosome:position), phenotype of interest, study in which it has been found significant, GWAS methods, group, alternative allele effect, standard error of the effect, lower bound of the 95% confidence interval for the allele effect, upper bound of the 95% confidence interval for the allele effect, sign of the allele effect.
**Results S4:** Region overlap between phenotypes are reported: the phenotype combination, the chromosome, the defined region start and stop, the positions of the first, second and, if they exist, third and fourth SNPs in the region, the loci found in this region, their start, stop position and strand information as well as their biotype.
**Results S5:** List of the SNPs in high LD ($R^{2}>0.8$) with SNPs identified as significant in at least one of our analyses. In detail, you can see the chromosome, name of the SNP identified as significant, its position, its closest locus, its location relative to its closest locus, the name of the SNP in high LD, its position, its closest locus, the $R^{2}$ value between these two SNPs, their distance from each other, the group, the phenotype of interest and the analysis in which the focal SNP has been found significant.
**Results S6:** Overlap between haplotype blocks found in Wragg et al. (2022) and SNPs identified as significant in our study. We can see the chromosome, the SNP name, its position, the start (BP1) and stop (BP2) position of the haplotype block, the size of the haplotype block, the number of SNPs found in the block by Wragg et al. (2022), in which analysis it has been found significant, the phenotype, the group, the number of genes in the block, their ID.
**Results S7:** Summary results of the GWAS performed with GEMMA are detailed: the sample sizes, the number of PC kept as covariates for GWAS with GEMMA, the $\lambda_{\mathrm{gc}}$ values for the QQplot, the PVE and their standard errors, the PGE and their standard errors, the estimated large sense heritability, rho estimates, number of variants with major effects, proportion of variants with non‐zero effect (and their 95% confidence intervals).
**Results S8:** Genetic correlations estimated using a Pearson test across phenotypes within groups and using a Spearman rank test within phenotypes across groups. The details for the correlation estimates, their *p* values and the 95% confidence intervals for the correlation estimate (in the case of Pearson’s correlation), the sign of the correlation for each of the GWAS methods, in each group and for each phenotype are presented.
**Results S9:** Mantel test performed to compare matrices are presented: the test statistics, the *p* values associated with different tests, and the 95% confidence interval borders as well as the signs of the Mantel correlation.
**Results S10:** Results for linear regression analysis for each phenotype, each group, to test the effect of environmental covariates such as the beekeeper, the apiary, the operator. We report the sum of squares, degrees of freedom, F value and *p* values for each model.
**Results S11:** Correlations between allele effects across phenotypes, groups and methods, estimated with Pearson’s, Spearman rank and linear regression.

## Data Availability

Raw sequences are made available under the bioproject accession PRJNA1083455 on NCBI (https://www.ncbi.nlm.nih.gov/bioproject?term=PRJNA1083455&cmd=DetailsSearch). Additional data: count files (from popoolation2), raw phenotypes, accession numbers for the sequences, summary statistics from GWAS analysis and scripts are available in the following repository: https://doi.org/10.57745/HHE4CZ. The scripts to perform the analysis are available on github (https://github.com/seynard/gwas_beestrong).

## References

[mec17637-bib-0001] Ament, S. A. , Q. W. Chan , M. M. Wheeler , et al. 2011. “Mechanisms of Stable Lipid Loss in a Social Insect.” Journal of Experimental Biology 214, no. 22: 3808–3821. 10.1242/jeb.060244. 22031746 PMC3202514

[mec17637-bib-0002] Aurori, C. M. , A. I. Giurgiu , B. H. Conlon , et al. 2021. “Juvenile Hormone Pathway in Honey Bee Larvae: A Source of Possible Signal Molecules for the Reproductive Behavior of *Varroa destructor* .” Ecology and Evolution 11, no. 2: 1057–1068. 10.1002/ece3.7125. 33520186 PMC7820148

[mec17637-bib-0003] Avalos, A. , M. Fang , H. Pan , et al. 2020. “Genomic Regions Influencing Aggressive Behavior in Honey Bees Are Defined by Colony Allele Frequencies.” Proceedings of the National Academy of Sciences 117: 17135–17141. 10.1073/pnas.1922927117. PMC7382227 32631983

[mec17637-bib-0004] Bernstein, R. , M. du , Z. G. du , A. S. Strauss , A. Hoppe , and K. Bienefeld . 2023. “First Large‐Scale Genomic Prediction in the Honey Bee.” Heredity 130: 1365–2540. 10.1038/s41437-023-00606-9. PMC10163272 36878945

[mec17637-bib-0005] Bernstein, R. , M. du , A. Hoppe , and K. Bienefeld . 2021. “Simulation Studies to Optimize Genomic Selection in Honey Bees.” Genetics Selection Evolution 53, no. 1: 64. 10.1186/s12711-021-00654-x. PMC8323320 34325663

[mec17637-bib-0006] Beye, M. , M. Hasselmann , M. K. Fondrk , R. E. Page Jr. , and S. W. Omholt . 2003. “The Gene Csd Is the Primary Signal for Sexual Development in the Honeybee and Encodes an SR‐Type Protein.” Cell 114, no. 4: 419–429. 10.1016/S0092-8674(03)00606-8. 12941271

[mec17637-bib-0007] Bienefeld, K. , and F. Pirchner . 1990. “Heritabilities for Several Colony Traits in the Honeybee ( *Apis mellifera carnica* ).” Apidologie 21, no. 3: 175–183. 10.1051/apido:19900302.

[mec17637-bib-0008] Bruckner, S. , M. Wilson , D. Aurell , et al. 2023. “A National Survey of Managed Honey Bee Colony Losses in the USA: Results From the Bee Informed Partnership for 2017–18, 2018–19, and 2019–20.” Journal of Apicultural Research 2: 1–15. 10.1080/00218839.2022.2158586.

[mec17637-bib-0009] Büchler, R. 2017. “Screening for Low Varroa Mite Reproduction (SMR) and Recapping in European Honey Bees.” *Research Network for Sustainable Bee Breeding*.

[mec17637-bib-0010] Büchler, R. , M. Kovačić , M. Buchegger , Z. Puškadija , A. Hoppe , and E. W. Brascamp . 2020. “Evaluation of Traits for the Selection of *Apis mellifera* for Resistance Against *Varroa destructor* .” Insects 11, no. 9: 618. 10.3390/insects11090618. 32927627 PMC7565760

[mec17637-bib-0011] Conlon, B. H. , A. Aurori , A. I. Giurgiu , et al. 2019. “A Gene for Resistance to the Varroa Mite (Acari) in Honey Bee ( *Apis mellifera* ) Pupae.” Molecular Ecology 28: 962–1083. 10.1111/mec.15080. 30916410

[mec17637-bib-0012] Cremer, S. , S. A. O. Armitage , and P. Schmid‐Hempel . 2007. “Social Immunity.” Current Biology 17, no. 16: R693–R702. 10.1016/j.cub.2007.06.008. 17714663

[mec17637-bib-0013] Dadoun, N. , M. Nait‐Mouloud , A. Mohammedi , and O. Sadeddine Zennouche . 2020. “Differences in Grooming Behavior Between Susceptible and Resistant Honey Bee Colonies After 13 Years of Natural Selection.” Apidologie 51, no. 5: 1297–9678. 10.1007/s13592-020-00761-6.

[mec17637-bib-0014] de Miranda, J. , L. Gauthier , M. Ribière , and Y. P. Chen . 2011. Honey Bee Viruses and Their Effect on Bee and Colony Health, 71–102. London, UK: Taylor and Francis.

[mec17637-bib-0015] Dietemann, V. , F. Nazzi , S. J. Martin , et al. 2012. “Standard Methods for Varroa Research.” Journal of Apicultural Research 52: 1–54. 10.3896/IBRA.1.52.1.09.

[mec17637-bib-0016] Dinno, A. 2009. “Implementing Horn's Parallel Analysis for Principal Component Analysis and Factor Analysis.” Stata Journal 9: 291–298. 10.1177/1536867X0900900207.

[mec17637-bib-0017] Dogantzis, K. A. , T. Tiwari , I. M. Conflitti , et al. 2021. “Thrice out of Asia and the Adaptive Radiation of the Western Honey Bee.” Science Advances 7, no. 49: eabj2151. 10.1126/sciadv.abj2151. 34860547 PMC8641936

[mec17637-bib-0018] Du, M. , R. Bernstein , A. Hoppe , and K. Bienefeld . 2022a. “Consequences of Incorrect Genetic Parameter Estimates for Single‐Trait and Multi‐Trait Genetic Evaluations in Honeybees.” Journal of Animal Breeding and Genetics 139, no. 6: 666–678. 10.1111/jbg.12728. 35775281

[mec17637-bib-0019] Du, M. , R. Bernstein , A. Hoppe , and K. Bienefeld . 2022b. “Influence of Model Selection and Data Structure on the Estimation of Genetic Parameters in Honeybee Populations.” G3: Genes, Genomes, Genetics 12: jkab450. 10.1093/g3journal/jkab450. 35100384 PMC8824827

[mec17637-bib-0020] Eynard, S. E. , C. Sann , B. Basso , et al. 2020. “Descriptive Analysis of the Varroa Non‐Reproduction Trait in Honey Bee Colonies and Association With Other Traits Related toVarroa Resistance.” Insects 11, no. 8: 492. 10.3390/insects11080492. 32752279 PMC7469219

[mec17637-bib-0021] Eynard, S. E. , A. Vignal , B. Basso , et al. 2022. “Reconstructing Queen Genotypes by Pool Sequencing Colonies in Eusocial Insects: Statistical Methods and Their Application to Honeybee.” Molecular Ecology Resources 22, no. 8: 3035–3048. 35816386 10.1111/1755-0998.13685 PMC9796407

[mec17637-bib-0022] Feldlaufer, M. F. , and K. Hartfelder . 1997. “Relationship of the Neutral Sterols and Ecdysteroids of the Parasitic Mite, *Varroa jacobsoni* to Those of the Honey Bee, *Apis mellifera* .” Journal of Insect Physiology 43, no. 6: 541–545. 10.1016/S0022-1910(97)00005-X. 12770416

[mec17637-bib-0023] Forêt, S. , and R. Maleszka . 2006. “Function and Evolution of a Gene Family Encoding Odorant Binding‐Like Proteins in a Social Insect, the Honey Bee ( *Apis mellifera* ).” Genome Research 16, no. 11: 1404–1413. 10.1101/gr.5075706. 17065610 PMC1626642

[mec17637-bib-0024] French, A. S. , K. L. Simcock , D. Rolke , S. E. Gartside , W. Blenau , and G. A. Wright . 2014. “The Role of Serotonin in Feeding and Gut Contractions in the Honeybee.” Journal of Insect Physiology 61, no. 100: 8–15. 10.1016/j.jinsphys.2013.12.005. 24374107 PMC3969292

[mec17637-bib-0025] Fries, I. , A. Imdorf , and P. Rosenkranz . 2006. “Survival of Mite Infested ( *Varroa destructor* ) Honey Bee ( *Apis mellifera* ) Colonies in a Nordic Climate.” Apidologie 37: 564–570. 10.1051/apido:2006031.

[mec17637-bib-0026] Gabel, M. , A. Hoppe , R. Scheiner , J. Obergfell , and R. Büchler . 2023. “Heritability of *Apis mellifera* Recapping Behavior and Suppressed Mite Reproduction as Resistance Traits Towards *Varroa destructor* .” Frontiers in Insect Science 3: 1135. 10.3389/finsc.2023.1135187 PMC10926398 38469460

[mec17637-bib-0027] Gebremedhn, H. , D. Claeys Bouuaert , M. Asperges , B. Amssalu , L. de Smet , and D. C. de Graaf . 2023. “Expression of Molecular Markers of Resilience Against *Varroa destructor* and Bee Viruses in Ethiopian Honey Bees ( *Apis mellifera simensis* ) Focussing on Olfactory Sensing and the RNA Interference Machinery.” Insects 14, no. 5: 436. 10.3390/insects14050436. 37233064 PMC10231090

[mec17637-bib-0028] Goulson, D. , E. Nicholls , C. Botías , and E. L. Rotheray . 2015. “Bee Declines Driven by Combined Stress From Parasites, Pesticides, and Lack of Flowers.” Science 347, no. 6229: 1255957. 10.1126/science.1255957. 25721506

[mec17637-bib-0029] Guarna, M. , A. P. Melathopoulos , E. Huxter , et al. 2015. “A Search for Protein Biomarkers Links Olfactory Signal Transduction to Social Immunity.” BMC Genomics 16: 63. 10.1186/s12864-014-1193-6. 25757461 PMC4342888

[mec17637-bib-0030] Guichard, M. , B. Dainat , S. Eynard , et al. 2021. “Identification of Quantitative Trait Loci Associated With Calmness and Gentleness in Honey Bees Using Whole‐Genome Sequences.” Animal Genetics 52: 472–481. 10.1111/age.13070. 33970494 PMC8360191

[mec17637-bib-0031] Guichard, M. , B. Dainat , S. Eynard , et al. 2022. “Two Quantitative Trait Loci Are Associated With Recapping of *Varroa destructor* ‐Infested Brood Cells in * Apis mellifera Mellifera* .” Animal Genetics 53: 1365–2052. 10.1111/age.13150. PMC9297925 34729804

[mec17637-bib-0032] Guichard, M. , V. Dietemann , M. Neuditschko , and B. Dainat . 2020. “Advances and Perspectives in Selecting Resistance Traits Against the Parasitic Mite *Varroa destructor* in Honey Bees.” Genetics Selection Evolution 52, no. 1: 71. 10.1186/s12711-020-00591-1. PMC7694340 33246402

[mec17637-bib-0033] Guichard, M. , B. Droz , E. W. Brascamp , A. von Virag , M. Neuditschko , and B. Dainat . 2021. “Exploring Two Honey Bee Traits for Improving Resistance Against *Varroa destructor* : Development and Genetic Evaluation.” Insects 12, no. 3: 216. 10.3390/insects12030216. 33802598 PMC8001962

[mec17637-bib-0034] Han, B. , J. Wu , Q. Wei , et al. 2024. “Life‐History Stage Determines the Diet of Ectoparasitic Mites on Their Honey Bee Hosts.” Nature Communications 15, no. 1: 725. 10.1038/s41467-024-44915-x. PMC10811344 38272866

[mec17637-bib-0035] Harbo, J. R. , and J. W. Harris . 1999. “Heritability in Honey Bees (Hymenoptera: Apidae) of Characteristics Associated With Resistance to *Varroa jacobsoni* (Mesostigmata: Varroidae).” Journal of Economic Entomology 92, no. 2: 261–265. 10.1093/jee/92.2.261.

[mec17637-bib-0036] Harbo, J. R. , and J. W. Harris . 2005. “Suppressed Mite Reproduction Explained by the Behavior of Adult Bees.” Journal of Apicultural Research 44, no. 1: 21–23. 10.1080/00218839.2005.11101141.

[mec17637-bib-0037] Harbo, J. R. , and J. W. Harris . 2009. “Responses to Varroa by Honey Bees With Different Levels of Varroa Sensitive Hygiene.” Journal of Apicultural Research 48, no. 3: 156–161. 10.3896/IBRA.1.48.3.02.

[mec17637-bib-0038] Hernandez, J. , A. Maisonnasse , M. Cousin , et al. 2020. “ColEval: Honeybee COLony Structure EVALuation for Field Surveys.” Insects 11, no. 1: 41. 10.3390/insects11010041. 31948048 PMC7023294

[mec17637-bib-0039] Hoppe, A. , R. Bernstein , M. Du , et al. 2022. “Heritability of Disease Resistance to Chronic Bee Paralysis, Chalkbrood and Nosemosis in the Honeybee (*A.M. Carnica*).” In: *Proceedings of 12th World Congress on Genetics Applied to Livestock Production (WCGALP)* (pp. 2556–2559). WageningenAcademic Publishers.

[mec17637-bib-0040] Hoppe, A. , M. Du , R. Bernstein , et al. 2020. “Substantial Genetic Progress in the International *Apis mellifera carnica* Population Since the Implementation of Genetic Evaluation.” Insects 11, no. 11: 768. 10.3390/insects11110768. 33171738 PMC7694995

[mec17637-bib-0041] Hu, H. , K. Bienefeld , J. Wegener , et al. 2016. “Proteome Analysis of the Hemolymph, Mushroom Body, and Antenna Provides Novel Insight Into Honeybee Resistance Against Varroa Infestation.” Journal of Proteome Research 15, no. 8: 2841–2854. 10.1021/acs.jproteome.6b00423. 27384112

[mec17637-bib-0042] Jacques, A. , M. Laurent , M. Ribière‐Chabert , et al. 2017. “A Pan‐European Epidemiological Study Reveals Honey Bee Colony Survival Depends on Beekeeper Education and Disease Control.” PLoS One 12, no. 3: 1–17. 10.1371/journal.pone.0172591. PMC5344352 28278255

[mec17637-bib-0043] Jiang, S. , T. Robertson , M. Mostajeran , et al. 2016. “Differential Gene Expression of Two Extreme Honey Bee ( *Apis mellifera* ) Colonies Showing Varroa Tolerance and Susceptibility.” Insect Molecular Biology 25, no. 3: 272–282. 10.1111/imb.12217. 26919127

[mec17637-bib-0044] Jones, J. C. , Z. G. du , R. Bernstein , et al. 2020. “Tool for Genomic Selection and Breeding to Evolutionary Adaptation: Development of a 100K Single Nucleotide Polymorphism Array for the Honey Bee.” Ecology and Evolution 10, no. 13: 6246–6256. 10.1002/ece3.6357. 32724511 PMC7381592

[mec17637-bib-0045] Josse, J. , and F. Husson . 2016. “missMDA: A Package for Handling Missing Values in Multivariate Data Analysis.” Journal of Statistical Software 70: 1–31. 10.18637/jss.v070.i01.

[mec17637-bib-0046] Klein, A. M. , B. E. Vaissière , J. H. Cane , et al. 2007. “Importance of Pollinators in Changing Landscapes for World Crops.” Proceedings of the Royal Society B: Biological Sciences 274: 303–313. 10.1098/rspb.2006.3721. PMC1702377 17164193

[mec17637-bib-0047] Kofler, R. , R. V. Pandey , and C. Schlötterer . 2011. “PoPoolation2: Identifying Differentiation Between Populations Using Sequencing of Pooled DNA Samples (Pool‐Seq).” Bioinformatics 27, no. 24: 3435–3436. 10.1093/bioinformatics/btr589. 22025480 PMC3232374

[mec17637-bib-0048] Le Conte, Y. , M. Ellis , and W. Ritter . 2010. “Varroa Mites and Honey Bee Health: Can Varroa Explain Part of the Colony Losses?” Apidologie 41, no. 3: 353–363. 10.1051/apido/2010017.

[mec17637-bib-0049] Lê, S. , J. Josse , and F. Husson . 2008. “FactoMineR: An R Package for Multivariate Analysis.” Journal of Statistical Software 25: 1–18. 10.18637/jss.v025.i01.

[mec17637-bib-0050] Li, H. 2013. “Aligning Sequence Reads, Clone Sequences and Assembly Contigs With BWAMEM.” In: *arXiv preprint arXiv:1303.3997*.

[mec17637-bib-0051] Li, H. , and R. Durbin . 2009. “Fast and Accurate Short Read Alignment With Burrows–Wheeler Transform.” Bioinformatics 25, no. 14: 1754–1760. 10.1093/bioinformatics/btp324. 19451168 PMC2705234

[mec17637-bib-0052] Liu, Y. , L. Yan , Z. Li , et al. 2016. “Larva‐Mediated Chalkbrood Resistance‐Associated Single Nucleotide Polymorphism Markers in the Honey Bee *Apis mellifera* .” Insect Molecular Biology 25, no. 3: 239–250. 10.1111/imb.12216. 26991518

[mec17637-bib-0053] Lorenz, S. 2016. “The Endangerment of Bees and New Developments in Beekeeping: A Social Science Perspective Using the Example of Germany.” International Journal of Environmental Studies 73, no. 6: 988–1005. 10.1080/00207233.2016.1220703.

[mec17637-bib-0054] Mam, B. , S. D. Karpe , and R. Sowdhamini . 2023. “Minus‐C Subfamily Has Diverged From Classic Odorant‐Binding Proteins in Honeybees.” Apidologie 54, no. 1: 12. 10.1007/s13592-022-00988-5.

[mec17637-bib-0055] Marta Guarna, M. , S. E. Hoover , E. Huxter , et al. 2017. “Peptide Biomarkers Used for the Selective Breeding of a Complex Polygenic Trait in Honey Bees.” Scientific Reports 7, no. 1: 8381. 10.1038/s41598-017-08464-2. 28827652 PMC5566959

[mec17637-bib-0056] McAfee, A. , A. Chapman , I. Iovinella , et al. 2018. “A Death Pheromone, Oleic Acid, Triggers Hygienic Behavior in Honey Bees ( *Apis mellifera* L.).” Scientific Reports 8, no. 1: 5719. 10.1038/s41598-018-24054-2. 29632403 PMC5890279

[mec17637-bib-0057] McLaren, W. , L. Gil , S. E. Hunt , et al. 2016. “The Ensembl Variant Effect Predictor.” Genome Biology 17, no. 1: 122. 10.1186/s13059-016-0974-4. 27268795 PMC4893825

[mec17637-bib-0058] Mondet, F. , C. Alaux , D. Severac , M. Rohmer , A. R. Mercer , and Y. le Conte . 2015. “Antennae Hold a Key to Varroa‐Sensitive Hygiene Behaviour in Honey Bees.” Scientific Reports 5, no. 1: 10454. 10.1038/srep10454. 26000641 PMC4441115

[mec17637-bib-0059] Mondet, F. , A. Beaurepaire , A. McAfee , et al. 2020. “Honey Bee Survival Mechanisms Against the Parasite *Varroa destructor* : A Systematic Review of Phenotypic and Genomic Research Efforts.” International Journal for Parasitology 50, no. 6: 433–447. 10.1016/j.ijpara.2020.03.005. 32380096

[mec17637-bib-0060] Mondet, F. , M. Parejo , M. D. Meixner , et al. 2020. “Evaluation of Suppressed Mite Reproduction (SMR) Reveals Potential for Varroa Resistance in European Honey Bees ( *Apis mellifera* L.).” Insects 11, no. 9: 595. 10.3390/insects11090595. 32899430 PMC7565386

[mec17637-bib-0061] Morris, A. P. 2011. “Transethnic Meta‐Analysis of Genomewide Association Studies.” Genetic Epidemiology 35: 809–822. 10.1002/gepi.20630. 22125221 PMC3460225

[mec17637-bib-0062] Noël, A. , Y. Le Conte , and F. Mondet . 2020. “ *Varroa destructor* : How Does It Harm *Apis mellifera* Honey Bees and What Can Be Done About It?” Emerging Topics in Life Sciences 1: 45–57. 10.1042/ETLS20190125. PMC7326341 32537655

[mec17637-bib-0063] Obšteter, J. , A. Marinč , J. Prešern , D. Wragg , and G. Gorjanc . 2022. “Inferring Whole‐Genome Tree Sequences and Population and Demographic Parameters of the Western Honeybee.” In: Proceedings of 12th World Congress on Genetics Applied to Livestock Production (WCGALP) (pp. 2552–2555). Wageningen Academic Publishers.

[mec17637-bib-0064] Oddie, M. , B. Dahle , and P. Neumann . 2018. “Reduced Postcapping Period in Honey Bees Surviving *Varroa destructor* by Means of Natural Selection.” Insects 9: 4. 30356021 10.3390/insects9040149 PMC6316798

[mec17637-bib-0065] Potts, S. G. , J. C. Biesmeijer , C. Kremen , P. Neumann , O. Schweiger , and W. E. Kunin . 2010. “Global Pollinator Declines: Trends, Impacts and Drivers.” Trends in Ecology and Evolution 25, no. 6: 345–353. 10.1016/j.tree.2010.01.007. 20188434

[mec17637-bib-0066] Pritchard, J. K. , M. Stephens , and P. Donnelly . 2000. “Inference of Population Structure Using Multilocus Genotype Data.” Genetics 155, no. 2: 945–959. 10835412 10.1093/genetics/155.2.945 PMC1461096

[mec17637-bib-0067] Purcell, S. , and C. Chang . 2015. “PLINK 1.9.” www.cog‐genomics.org/plink/1.9/.

[mec17637-bib-0068] Purcell, S. , B. Neale , K. Todd‐Brown , et al. 2007. “PLINK: A Tool Set for Whole‐Genome Association and Population‐Based Linkage Analyses.” American Journal of Human Genetics 81, no. 3: 559–575. 10.1086/519795. 17701901 PMC1950838

[mec17637-bib-0069] Rinderer, T. E. , J. W. Harris , G. J. Hunt , and L. I. de Guzman . 2010. “Breeding for Resistance to *Varroa destructor* in North America.” Apidologie 41, no. 3: 409–424. 10.1051/apido/2010015.

[mec17637-bib-0070] Rinkevich, F. D. 2020. “Detection of Amitraz Resistance and Reduced Treatment Efficacy in the Varroa Mite, *Varroa destructor* , Within Commercial Beekeeping Operations.” PLoS One 15: 1–12. 10.1371/journal.pone.0227264. PMC6968863 31951619

[mec17637-bib-0071] Rosenkranz, P. , P. Aumeier , and B. Ziegelmann . 2010. “Biology and Control of *Varroa destructor* .” Journal of Invertebrate Pathology 103: S96–S119. 19909970 10.1016/j.jip.2009.07.016

[mec17637-bib-0072] Rothenbuhler, W. C. 1964. “Behavior Genetics of Nest Cleaning in Honey Bees. IV. Responses of F1 and Backcross Generations to Disease‐Killed Blood.” American Zoologist 4: 111–123. 10.1093/icb/4.2.111. 14172721

[mec17637-bib-0073] Ryabov, E. V. , G. R. Wood , J. M. Fannon , et al. 2014. “A Virulent Strain of Deformed Wing Virus (DWV) of Honeybees ( *Apis mellifera* ) Prevails After *Varroa destructor* ‐Mediated, or In Vitro, Transmission.” PLoS Pathogens 10, no. 6: e1004230. 10.1371/journal.ppat.1004230. 24968198 PMC4072795

[mec17637-bib-0074] Saelao, P. , M. Simone‐Finstrom , A. Avalos , et al. 2020. “Genome‐Wide Patterns of Differentiation Within and Among U.S. Commercial Honey Bee Stocks.” BMC Genomics 21, no. 1: 704. 10.1186/s12864-020-07111-x. 33032523 PMC7545854

[mec17637-bib-0075] Shorter, J. R. , M. Arechavaleta‐Velasco , C. Robles‐Rios , and G. J. Hunt . 2012. “A Genetic Analysis of the Stinging and Guarding Behaviors of the Honey Bee.” Behavior Genetics 42, no. 4: 663–674. 10.1007/s10519-012-9530-5. 22327626

[mec17637-bib-0076] Southey, B. R. , P. Zhu , M. K. Carr‐Markell , et al. 2016. “Characterization of Genomic Variants Associated With Scout and Recruit Behavioral Castes in Honey Bees Using Whole‐Genome Sequencing.” PLoS One 11, no. 1: e0146430. 10.1371/journal.pone.0146430. 26784945 PMC4718678

[mec17637-bib-0077] Speed, D. , N. Cai , M. R. Johnson , S. Nejentsev , and D. J. Balding . 2017. “Reevaluation of SNP Heritability in Complex Human Traits.” Nature Genetics 49, no. 7: 986–992. 10.1038/ng.3865. 28530675 PMC5493198

[mec17637-bib-0078] Speed, D. , G. Hemani , M. R. Johnson , and D. J. Balding . 2012. “Improved Heritability Estimation From Genome‐Wide SNPs.” American Journal of Human Genetics 91, no. 6: 1011–1021. 10.1016/j.ajhg.2012.10.010. 23217325 PMC3516604

[mec17637-bib-0079] Spivak, M. , and R. G. Danka . 2020. “Perspectives on Hygienic Behavior in Apis Mellifera and Other Social Insects.” Apidologie 52: 1297–9678. 10.1007/s13592-020-00784-z.

[mec17637-bib-0080] Stephens, M. 2017. “False Discovery Rates: A New Deal.” Biostatistics 18, no. 2: 275–294. 10.1093/biostatistics/kxw041. 27756721 PMC5379932

[mec17637-bib-0081] Tarpy, D. R. , S. Hatch , and D. J. C. Fletcher . 2000. “The Influence of Queen Age and Quality During Queen Replacement in Honeybee Colonies.” Animal Behaviour 59, no. 1: 97–101. 10.1006/anbe.1999.1311. 10640371

[mec17637-bib-0082] Techer, M. A. , R. V. Rane , M. L. Grau , et al. 2019. “Divergent Evolutionary Trajectories Following Speciation in Two Ectoparasitic Honey Bee Mites.” Communications Biology 2, no. 1: 357. 31583288 10.1038/s42003-019-0606-0 PMC6773775

[mec17637-bib-0083] Teixeira, E. W. , R. M. de Paiva Daibert , L. A. Glatzl Júnior , et al. 2021. “Transcriptomic Analysis Suggests Candidate Genes for Hygienic Behavior in African‐Derived *Apis mellifera* Honeybees.” Apidologie 52: 1297–9678. 10.1007/s13592-020-00834-6.

[mec17637-bib-0084] Traynor, K. S. , F. Mondet , J. R. de Miranda , et al. 2020. “ *Varroa destructor* : A Complex Parasite, Crippling Honey Bees Worldwide.” Trends in Parasitology 36, no. 7: 592–606. 10.1016/j.pt.2020.04.004. 32456963

[mec17637-bib-0085] Tsuruda, J. M. , J. W. Harris , L. Bourgeois , R. G. Danka , and G. J. Hunt . 2012. “High‐Resolution Linkage Analyses to Identify Genes That Influence Varroa Sensitive Hygiene Behavior in Honey Bees.” PLoS One 7, no. 11: e48276. 10.1371/journal.pone.0048276. 23133626 PMC3487727

[mec17637-bib-0086] Urbut, S. M. , G. Wang , P. Carbonetto , and M. Stephens . 2019. “Flexible Statistical Methods for Estimating and Testing Effects in Genomic Studies With Multiple Conditions.” Nature Genetics 51, no. 1: 187–195. 10.1038/s41588-018-0268-8. 30478440 PMC6309609

[mec17637-bib-0087] Uzunov, A. , E. W. Brascamp , M. Du , and R. Büchler . 2022. “Initiation and Implementation of Honey Bee Breeding Programs.” Bee World 99, no. 2: 50–55. 10.1080/0005772X.2022.2031545.

[mec17637-bib-0088] Verlinden, H. , R. Vleugels , and J. Vanden Broeck . 2015. “Serotonin, Serotonin Receptors and Their Actions in Insects.” Neurotransmitter 2: e314. 10.14800/nt.314.

[mec17637-bib-0089] von Virag, A. , M. Guichard , M. Neuditschko , V. Dietemann , and B. Dainat . 2022. “Decreased Mite Reproduction to Select *Varroa destructor* (Acari: Varroidae) Resistant Honey Bees (Hymenoptera: Apidae): Limitations and Potential Methodological Improvements.” Journal of Economic Entomology 115, no. 3: 695–705. 10.1093/jee/toac022. 35380682 PMC9175287

[mec17637-bib-0090] Wallberg, A. , I. Bunikis , O. V. Pettersson , et al. 2019. “A Hybrid De Novo Genome Assembly of the Honeybee, *Apis mellifera* , With Chromosome‐Length Scaffolds.” BMC Genomics 20, no. 1: 275. 10.1186/s12864-019-5642-0. 30961563 PMC6454739

[mec17637-bib-0091] Wang, X. , H. X. Chua , P. Chen , et al. 2013. “Comparing Methods for Performing Trans‐Ethnic Meta‐Analysis of Genome‐Wide Association Studies.” Human Molecular Genetics 22, no. 11: 2303–2311. 10.1093/hmg/ddt064. 23406875

[mec17637-bib-0092] Woyke, J. 1963. “Drones From Fertilized Eggs and the Biology of Sex Determination in the Honey Bee, Apimondia, Prague 12‐17.08.1963.” Bulletin de l'Académie Polonaise Des Sciences 9: 251–254. 10.13140/2.1.4890.8161.

[mec17637-bib-0093] Wragg, D. , S. E. Eynard , B. Basso , et al. 2022. “Complex Population Structure and Haplotype Patterns in the Western European Honey Bee From Sequencing a Large Panel of Haploid Drones.” Molecular Ecology Resources 22, no. 8: 3068–3086. 35689802 10.1111/1755-0998.13665 PMC9796960

[mec17637-bib-0094] Zayed, A. 2004. “Effective Population Size in Hymenoptera With Complementary Sex Determination.” Heredity 93, no. 6: 627–630. 10.1038/sj.hdy.6800588. 15354193

[mec17637-bib-0095] Zhou, X. , P. Carbonetto , and M. Stephens . 2013. “Polygenic Modeling With Bayesian Sparse Linear Mixed Models.” PLoS Genetics 9, no. 2: e1003264. 10.1371/journal.pgen.1003264. 23408905 PMC3567190

[mec17637-bib-0096] Zhou, X. , and M. Stephens . 2012. “Genome‐Wide Efficient Mixed‐Model Analysis for Association Studies.” Nature Genetics 44, no. 7: 821–824. 22706312 10.1038/ng.2310 PMC3386377

