# Supplementary material for: Sequence‐Based Multi Ancestry Association Study Reveals the Polygenic Architecture of Varroa destructor Resistance in the Honeybee Apis mellifera
Source: Mol Ecol. 2024 Dec 31;34(3):e17637. doi: 10.1111/mec.17637 (PMC11754705; doi:10.1111/mec.17637)
Supplement: Supplementary file 1 — Figure S1: QQplot for the GWAS on each group and phenotype. QQplot representing the observed −log10(p values) as function of the expected −log10(p values) for each of the GWAS performed with GEMMA for the three groups A. m. ligustica & carnica, A. m. mellifera and hybrids, for the three phenotypes of interest, varroa infestation (varroa_inf), MNR and recapping (recap). Parameters λgc calculated for each of these analyses. Methods S1: Details on phenotypes and statistical transformations applied for association study. Methods S2: Details on the genomic relationship matrix estimations and description of the SNP weights estimated by LDAK. [file MEC-34-e17637-s001.pdf]

Sequence-based multi ancestry genome-wide association studies reveal the polygenic architecture of *Varroa destructor* resistance in Western honey bees *Apis mellifera*

Eynard et al.

Corresponding authors: sonia.eynard@inrae.fr, bertrand.servin@inrae.fr

1 Details on phenotypes and statistical transformations applied for association study

1.1 Statistical transformation

In order to fit the assumption of Normality underlying genome wide associations models all of our phenotypes of interest were corrected. These phenotypes are of three types: (i) link to varroa infestation *varroa_inf*, (ii) mite non reproduction *MNR* and (iii) recapping behaviour *recap*.

1.1.1 Varroa infestation

Varroa infestation was quantified with four different measures: phoretic infestation (on adult bees, using two different methods), brood infestation, and total mite load.

Phoretic varroa (*v_pho*) was transformed as log(x+1) to better account for 0 values. A transformation using the 4th root was applied to varroa mitochondria ratio (*v_mito*). For the brood cell varroa infestation (*v_brood*) we used the posterior mean of a beta binomial model with a uniform distribution as prior. This transformation allows to weigh (shrink) estimations based on the number of infested brood cells, which can be quite heterogeneous due to variation in the overall infestation of the colony. A logit transformation was applied thereafter to reduce bias towards low values. Finally, a transformation using the 4th root was also applied to varroa load (*v_load*).

1.1.2 Recapping of infested cells

As for the brood cell varroa infestation, and for the same reason of heterogeneity of infestation levels across colonies, the posterior mean of a beta binomial model with a uniform distribution as prior, followed by logit transformation, was used for the recapping estimates (*recap*).

1.1.3 Mite non reproduction

Finally, for *MNR*, the empirical Bayes estimate, proposed by Mondet et al. 2020 [1] and Eynard et al. 2020 [2], was used.

1.1.4 Phenotype combination

The phenotypes linked to varroa infestation were highly correlated, and explained the first axis of the Principal Component Analysis (PCA) on phenotypes (Figure 1) and thereafter called *varroa_inf*.

Tab. 1: **Summary of the phenotypes used in this study, the statistical transformations applied and their combination**

**Initial name**	**Phenotypes description**	**Transformation**	**Combination**	**Final name**
v_pho	Phoretic varroa	log(x+1)		
v_brood	Varroa in brood cells	Posterior mean + logit		
v_load	Colony varroa load	4th root	PCA	*Varroa_inf*
v_mito	Sequencing depth varroa mitochondria / honey bee	4th root		
MNR	Mite non reproduction	Empirical Bayes	none	*MNR*
recap	Recapping	Posterior mean + logit	none	*Recap*

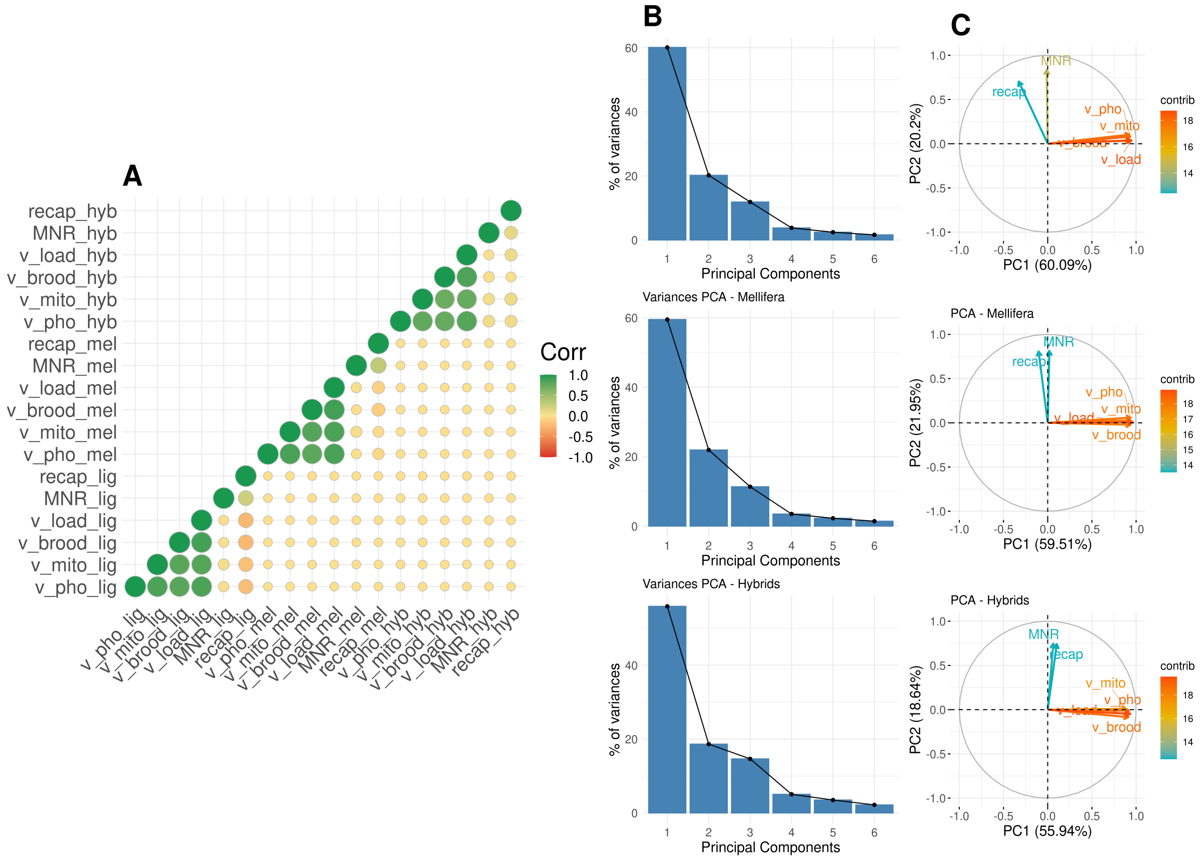

Fig. 1: **Correlation and principal component analysis.** Description of the correlation between phenotypes and PCA. **(A)** shows the correlations between our original phenotypes, with larger dots for stronger, green for positive and red for negative correlations. **(B)** summarises the percentage of variance explained by each of the PCs from axis 1 to 5. **(C)** shows our phenotypes on PCA for axis 1, 2 and 3, the colour gives the contribution of each variable to the axis, with red as the highest. The correlation and PCA estimates are detailed for each of the three groups defined in our study, *A. m. ligustica & carnica*, *A. m. mellifera* and the hybrids (n=1,423).

After transformation, the three main phenotypes in each of the groups showed a distribution close to a Normal distribution (Figure 2).

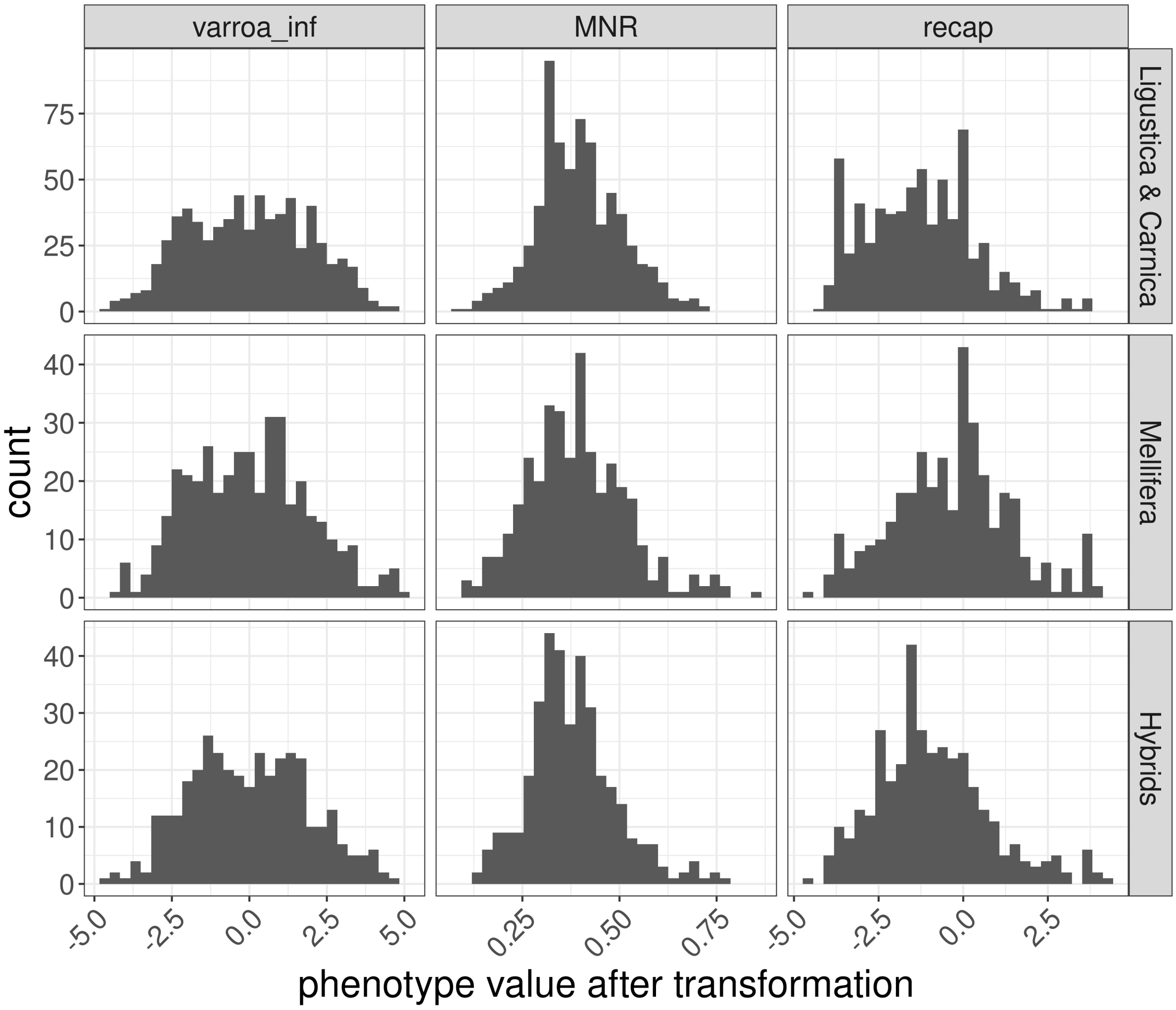

Fig. 2: **Distribution of the phenotypes.** Distribution of the three phenotypes used for the Genome wide association study across the three groups.

The number of colonies phenotyped for each of the traits of interest is summarised in Table 2.

Tab. 2: **Number of samples per group**

	**Sequenced**	**Varroa infestation**					**Resistance to varroa**	
		v_pho	***v_mito***	***v_brood***	***v_load***	***Varroa_inf***	***MNR***	***Recap***
Ligustica & Carnica	703	634	632	667	626	669	667	667
Mellifera	407	397	319	357	351	397	357	357
Hybrids	382	355	332	356	350	357	356	356

1.2 Two methods to measure phoretic infestation

Phoretic infestation was measured in two different ways:

(i) as the count of phoretic varroa for a sample of about 300 adult bees, brought back to a count for 100 bees, following the standard detergent method [3], thereafter called *v_pho*.

(ii) using the depth of varroa mitochondrial DNA sequencing from pool sequencing, relative to the depth of sequencing of honey bees, as a proxy for the number of varroa found in the adult bee pool sampled, thereafter called *v_mito*.

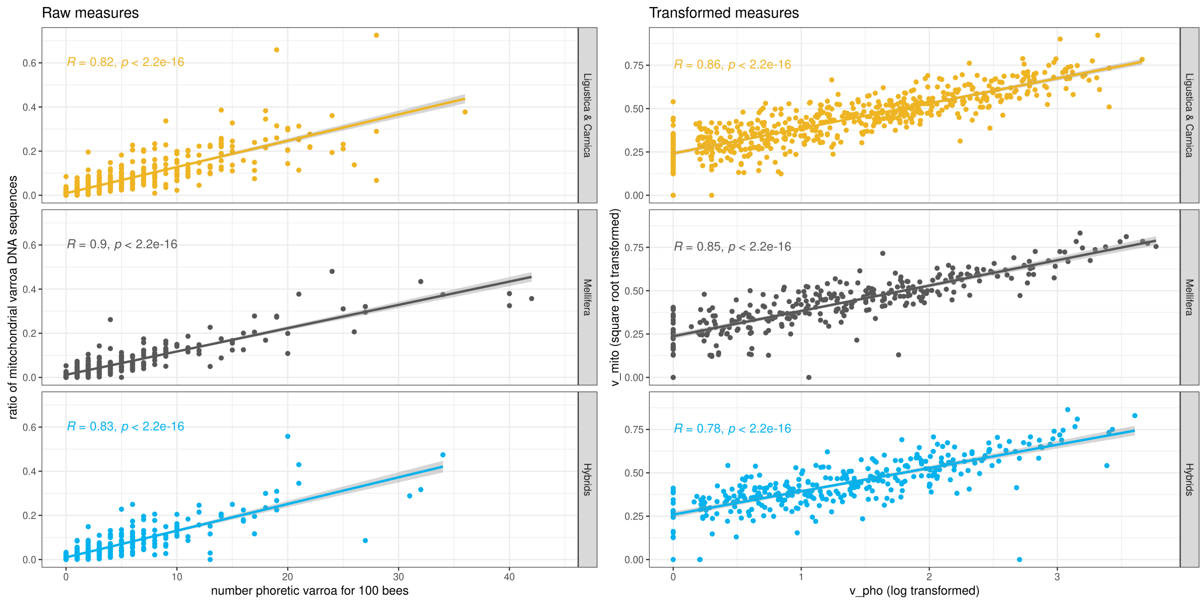

Fig. 3: **Correlation between the raw phoretic varroa count and the ratio of varroa mitochondrial DNA and between *v_pho* and *v_mito*, after transformation to follow the normal distribution.** The correlations are estimated for each of the three groups. Correlation coefficient R and its p-value are presented.

We can see that the two estimates for phoretic varroa, raw or transformed, are highly correlated ($R > 0.8$) (Figure 3). It can be noted that for some colonies no phoretic varroa are observed when they are actually measured when sequencing. Using the sequencing rate of varroa mitochondria seems to be a good alternative to phoretic varroa count. This method would provide a phenotype in the same sample as the sequencing, without asking for a dedicated measurement performed by the beekeeper. Unarguably, *v_mito* should be a phenotype of interest for large scale studies focusing on varroa infestation in honey bee colonies.

2 Details on the genomic relationship matrix estimations and description of the SNP weights estimated by LDAK

2.1 Genomic relationship matrices

Genomic relationship matrices (GRM) were estimated on data from pool sequencing, thus on allele frequencies. For each of the three groups defined based on their genetic backgrounds, after filtering for SNPs with minor allele frequency (MAF) above 0.01 and missing rate below 5% we retained 2,832,721; 2,413,514 and 2,912,182 markers for *A. m. ligustica & carnica*, *A. m. mellifera* and the hybrids respectively. GRM estimations, using the program LDAK with weighted SNPs [4], was performed on 292,992; 282,440 and 254,891 SNPs for *A. m. ligustica & carnica*, *A. m. mellifera* and the hybrids respectively, which is about 10% of the set of the total number of SNPs (Table 3).

Tab. 3: **Number of SNPs used per group after filtering**

	**Pool seq**	**GRM LDAK**
Ligustica & Carnica	2,832,721	292,922
Mellifera	2,413,514	282,440
Hybrids	2,912,182	254,891

In brief, when using LDAK, weights are given to SNPs based on linkage disequilibrium (Figure 4). Knowing that the honey bee genome is about 250Mb, that we have about 2.5 million SNPs for this genome and that only 10% of them are kept for GRM estimation after weighing by LDAK we can hypothesise that linkage disequilibrium is lost after 1Kb. Only SNPs having a non-null weight are retained for further GRM estimations. The GRM estimated based on a subset of relevant SNPs seem to better capture the population structure seen in the sample than traditional methods, such as GEMMA [5], when using data like allele frequencies from pool sequencing.

We can observe this pattern when looking at GRM estimates based on allele frequencies or reconstructed queen genotypes for each of our groups independently or all of the colonies together (Figure 5, 6, 7, 8).

When looking at the heatmaps built from the GRM estimations we can see that in all cases standard estimates, as provided by GEMMA [5], allow to distinguish between genetic backgrounds, and large group structures (mostly in correlation with beekeepers' association memberships), whereas estimations provided by LDAK [4] further discriminate fine structure within each group allowing to better take into account fine within beekeepers' association distinction, for example at the individual beekeeper level. Using GRM estimated with LDAK

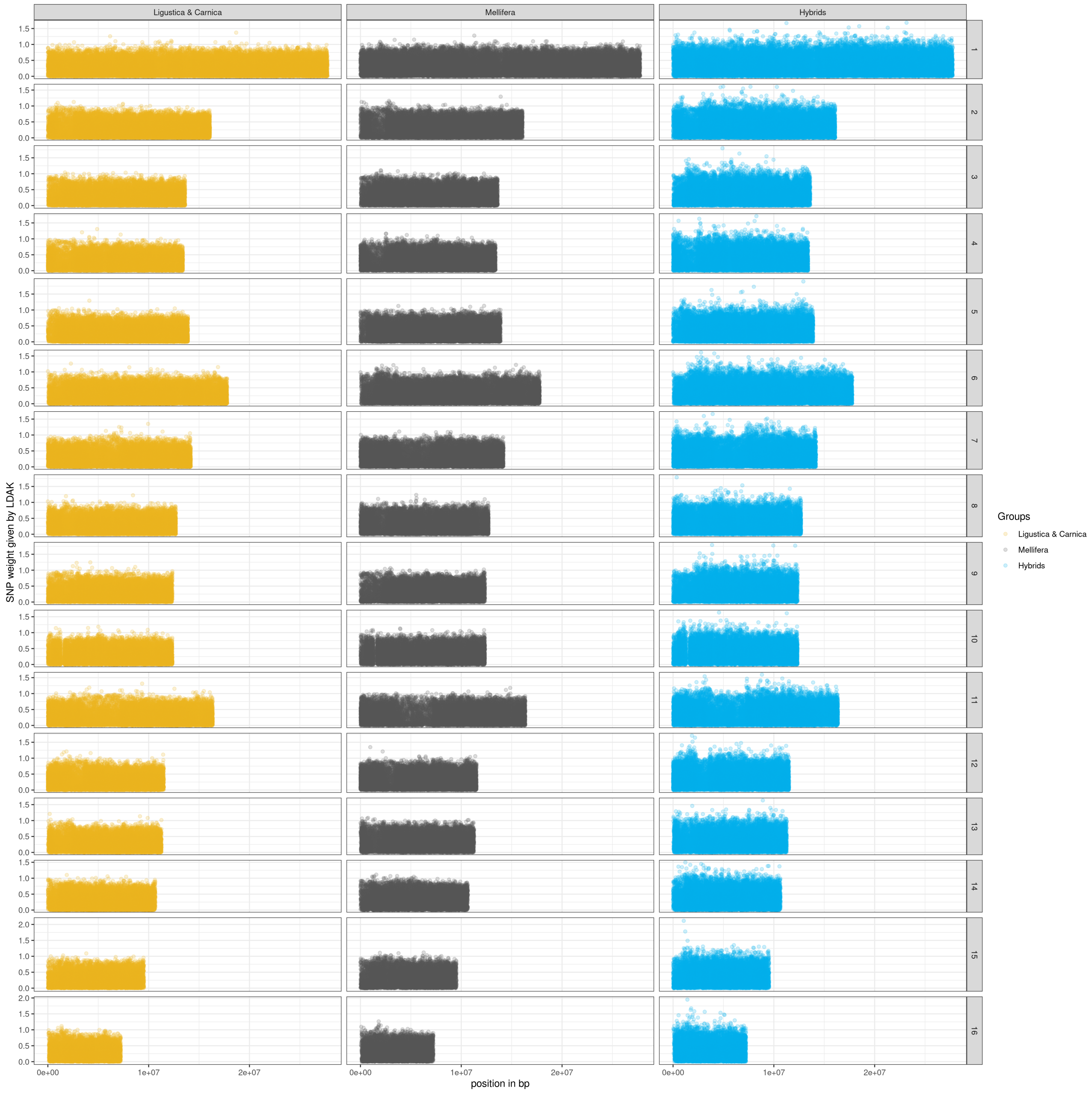

Fig. 4: **Values of SNP weights along the genome** Weights given to each SNPs by LDAK, along the genome, for each of the three groups.

allowed us to better account for fine population structure in our genome wide association studies (GWAS).

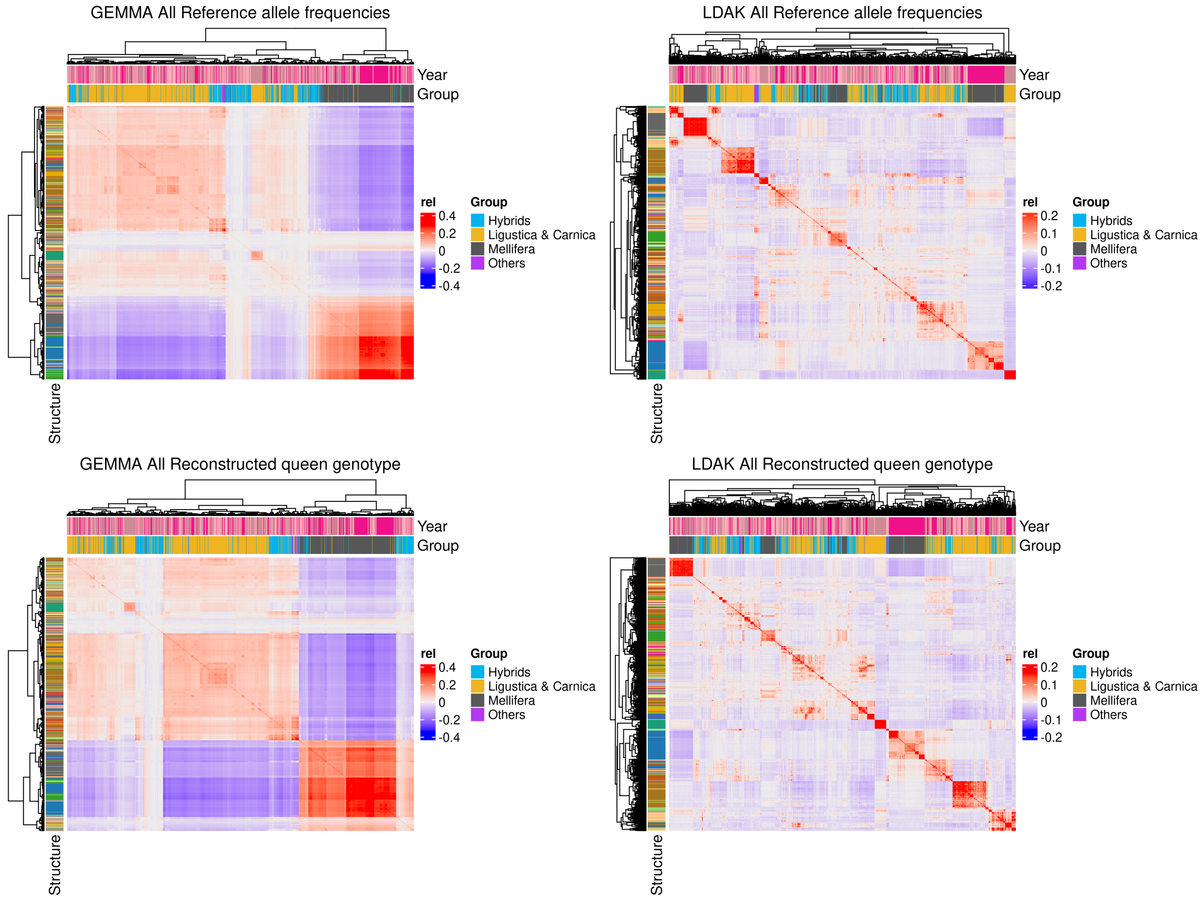

Fig. 5: **Genomic relationship matrices for all colonies** GRM estimated from allele frequencies or reconstructed queen genotypes, using LDAK or GEMMA, for all the colonies.

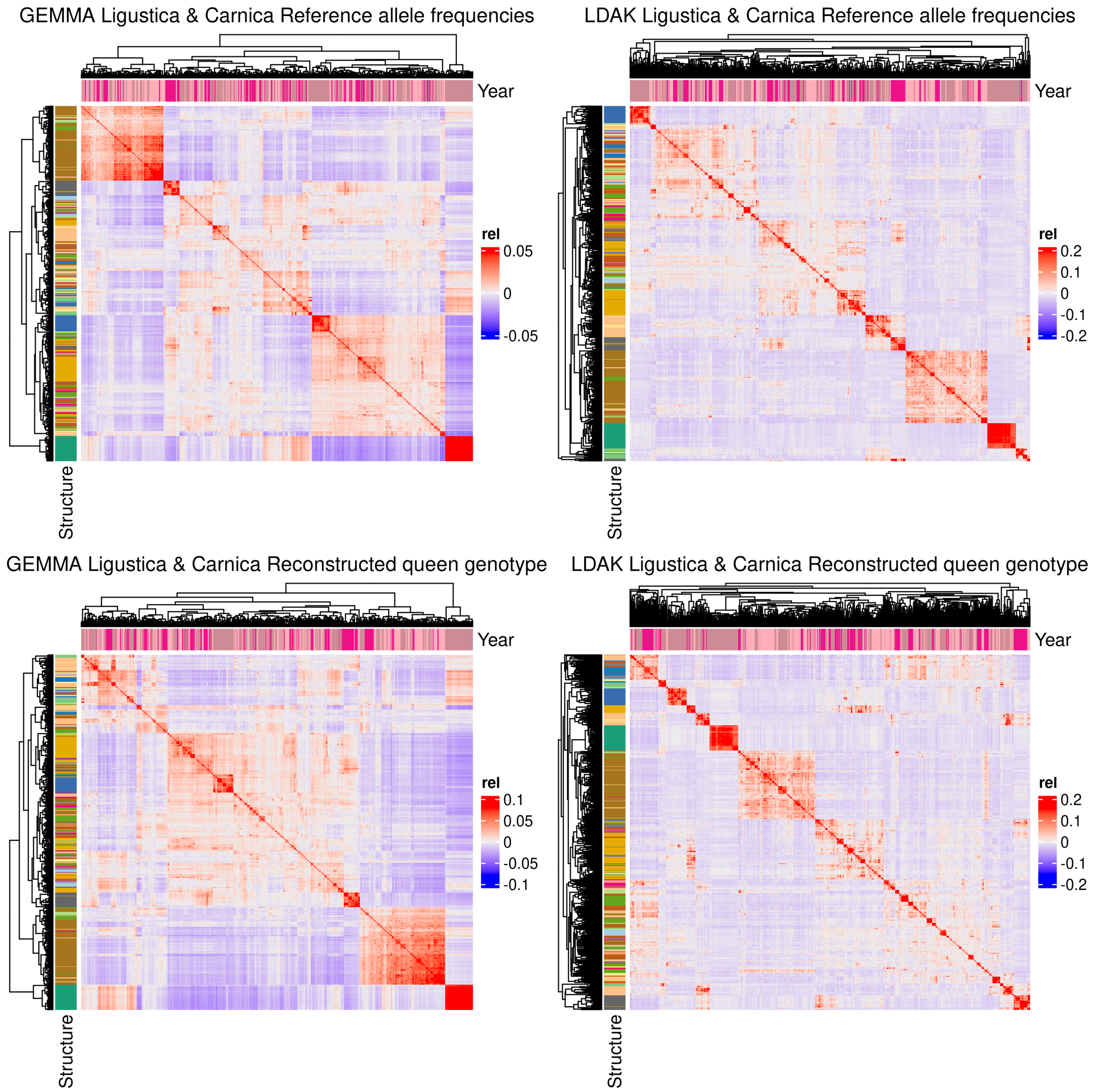

Fig. 6: **Genomic relationship matrices for Ligustica & Carnica** GRM estimated from allele frequencies or reconstructed queen genotypes, using LDAK or GEMMA, for the colonies in the ligustica & carnica group.

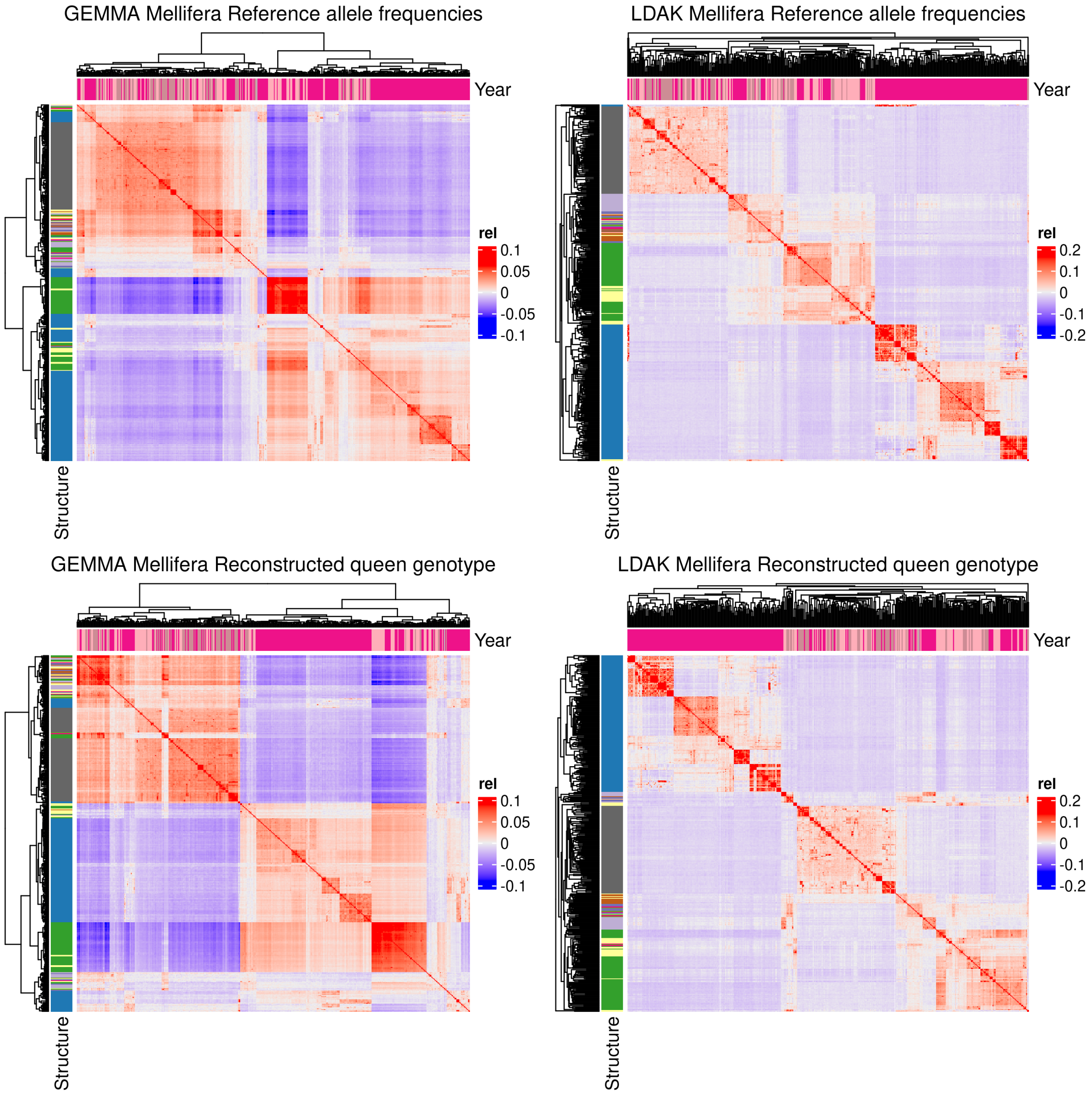

Fig. 7: **Genomic relationship matrices for Mellifera** GRM estimated from allele frequencies or reconstructed queen genotypes, using LDAK or GEMMA, for the colonies in the mellifera group.

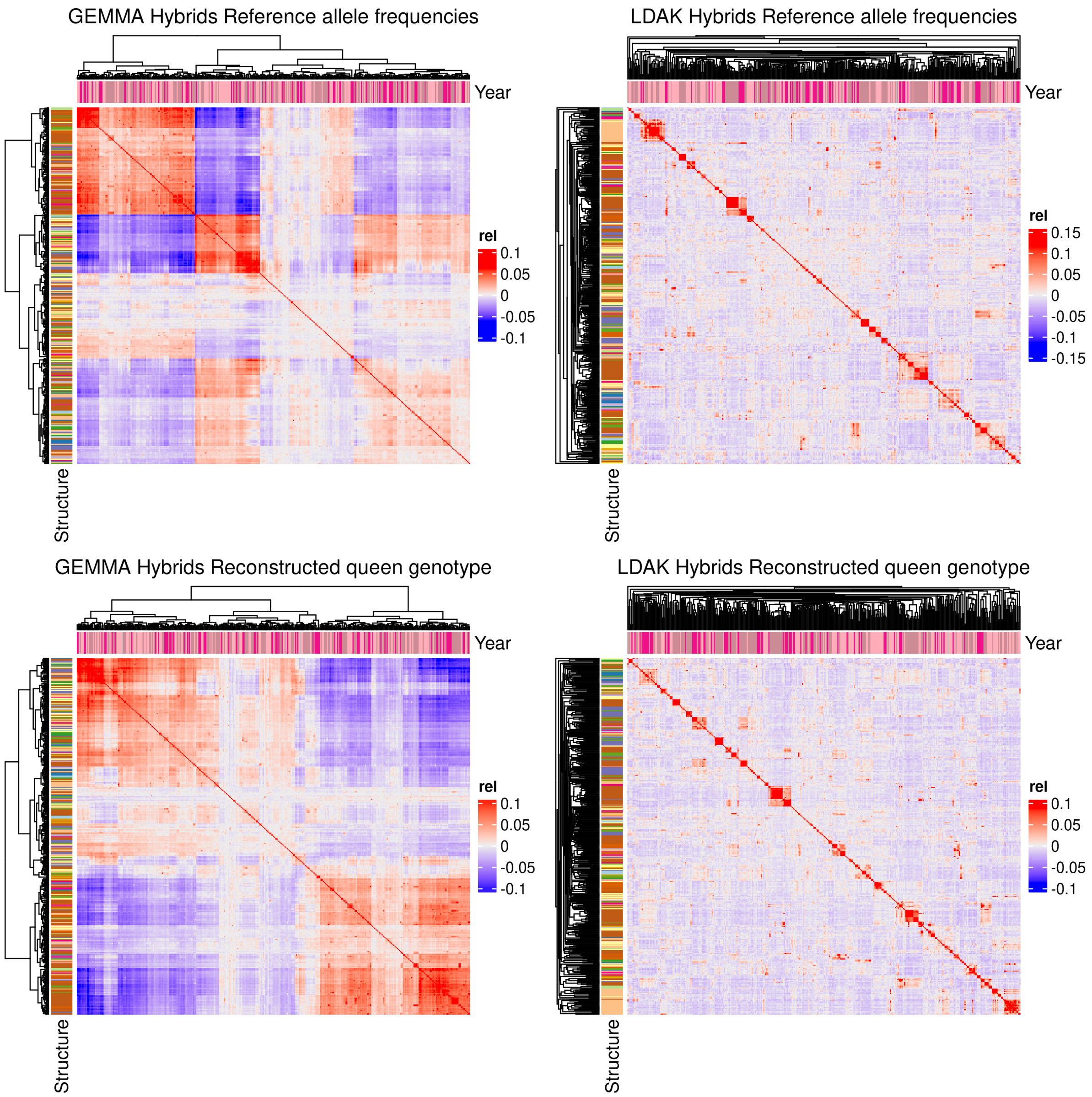

Fig. 8: **Genomic relationship matrices for hybrids** GRM estimated from allele frequencies or reconstructed queen genotypes, using LDAK or GEMMA, for the colonies in the hybrid group.

References

[1] F. Mondet et al. “Evaluation of Suppressed Mite Reproduction (SMR) Reveals Potential for Varroa Resistance in European Honey Bees (*Apis mellifera L.*)” In: *Insects* 11.9 (2020). ISSN: 2075-4450. DOI: 10.3390/insects11090595.

[2] S. E. Eynard et al. “Descriptive Analysis of the Varroa Non-Reproduction Trait in Honey Bee Colonies and Association with Other Traits Related to Varroa Resistance”. In: *Insects* 11.8 (2020). ISSN: 2075-4450 (Print) 2075-4450. DOI: 10.3390/insects11080492.

[3] V. Dietemann et al. “Standard methods for varroa research”. In: *Journal of Apicultural Research* 52 (2012), pp. 1–54. DOI: 10.3896/IBRA.1.52.1.09.

[4] D. Speed et al. “Reevaluation of SNP heritability in complex human traits”. In: *Nature Genetics* 49.7 (2017), pp. 986–992. ISSN: 1546-1718. DOI: 10.1038/ng.3865. URL: https://doi.org/10.1038/ng.3865.

[5] X. Zhou and M. Stephens. “Genome-wide efficient mixed-model analysis for association studies”. In: *Nature genetics* 44.7 (2012), pp. 821–824. URL: https://www.ncbi.nlm.nih.gov/pmc/PMC3386377/.
